# Ocular gene therapy targeting retinal angiogenesis and vascular leakage: translational and clinical evidence from a systematic review and meta-analysis

**DOI:** 10.1007/s10456-026-10050-y

**Published:** 2026-08-31

**Authors:** Kai-Yang Chen, Hoi-Chun Chan, Chi-Ming Chan

**Affiliations:** 1 Department of General Medicine, Chang Gung Memorial Hospital (Linkou Branch), Taoyuan, Taiwan; 2 School of Pharmacy, China Medical University, Taichung, Taiwan; 3 Department of Ophthalmology, Cardinal Tien Hospital, New Taipei City, Taiwan; 4 School of Medicine, Fu Jen Catholic University, New Taipei City, Taiwan

**Keywords:** Ocular gene therapy, Adeno-associated virus, Neovascularization, Vascular permeability, Retinal disease, Meta-analysis, Safety, Efficacy

## Abstract

**Introduction:**

Retinal diseases driven by pathological angiogenesis and vascular leakage, including neovascular age-related macular degeneration and diabetic retinopathy, impose substantial visual and treatment burdens because current anti-vascular endothelial growth factor (anti-VEGF) therapy requires repeated intravitreal administration. Ocular gene therapy can provide durable intraocular expression of therapeutic proteins and sustained pathway-level disease control.

**Methods:**

This systematic review and meta-analysis was prospectively registered in the International Prospective Register of Systematic Reviews (PROSPERO) and conducted according to the Preferred Reporting Items for Systematic Reviews and Meta-Analyses (PRISMA) 2020 guidelines. PubMed, Scopus, Web of Science, ScienceDirect, and the Cochrane Library were searched from inception to April 21, 2026. Eligible studies included preclinical, in vitro, and clinical investigations of gene-based interventions targeting retinal angiogenesis, vascular permeability, or related anatomical and treatment-burden outcomes. Random-effects meta-analyses were performed using standardized mean differences (SMDs), mean differences (MDs), and logit event rates.

**Results:**

Twenty-five studies were included. Preclinical gene therapy significantly reduced pathological neovascularization, with a pooled standardized mean difference (SMD) of −1.16 (95% confidence interval [CI], −1.48 to −0.84; p < 0.001) and negligible heterogeneity (I² = 0%). Vascular leakage was also significantly reduced across preclinical experimental studies, with a pooled mean difference (MD) of 0.215 (95% CI, 0.178–0.251; p < 0.001; I^2^ = 0%) after directional harmonization. In clinical cohorts, ocular gene therapy significantly reduced central subfield thickness (CST) by −55.30 μm (95% CI, −72.58 to −38.03; p < 0.001) and was associated with reduced anti-vascular endothelial growth factor (anti-VEGF) treatment burden, with a pooled logit event rate of 0.62 (95% CI, 0.20–1.04; p = 0.0039), corresponding to a pooled proportion of approximately 65% of participants meeting study-specific criteria for reduced supplemental anti-VEGF use.

**Conclusion:**

Ocular gene therapy shows strong preclinical anti-angiogenic and anti-permeability effects, with emerging clinical evidence of anatomical improvement and reduced anti-VEGF treatment burden in neovascular retinal disease cohorts. Larger disease-specific randomized trials are required to confirm long-term efficacy, safety, and patient selection.

**Supplementary Information:**

The online version contains supplementary material available at https://doi.org/10.1007/s10456-026-10050-y.

## Introduction

Retinal vascular and neovascular disorders represent a major cause of irreversible visual impairment worldwide and share convergent final common pathways that include pathological angiogenesis, breakdown of the vascular barrier with macular edema, and progressive neurovascular degeneration. Among these conditions, diabetic retinopathy (DR) serves as a high burden exemplar of retinal microvascular disease. Globally, DR affects approximately 22.27% of individuals with diabetes, corresponding to about 103.12 million adults in 2020, and is projected to increase to 160.50 million by 2045. Vision threatening DR accounts for 6.17% of individuals with diabetes and is expected to rise from 28.54 million in 2020 to 44.82 million by 2045 [1, 2, 3, 38]. Marked regional variation exists, with the highest prevalence reported in Africa (35.90%) and North America (33.30%) and the lowest in South and Central America (13.37%). Notably, the same angiogenic and permeability-driven mechanisms are also central to neovascular age-related macular degeneration (nAMD) and choroidal neovascularization (CNV), providing a biological rationale to integrate evidence across retinal angiogenic indications [4]. 

These processes culminate in capillary nonperfusion, tissue hypoxia, and compensatory neovascularization, ultimately driving retinal damage and vision loss. Importantly, vascular endothelial growth factor (VEGF)-driven pathological angiogenesis and concomitant blood-retinal barrier failure represent shared hallmarks across ischemic retinopathies and neovascular retinal diseases, supporting the translational relevance of therapeutic strategies that modulate VEGF signaling and endothelial stability across multiple retinal indications [4, 5]. DR imposes a substantial economic burden from a global perspective. Diabetic eye disease imposes substantial direct and indirect costs worldwide. In low- and middle-income countries, vision-threatening DR is more prevalent because of limited screening, restricted access to treatment, and reduced awareness. In Jordan, diabetic retinopathy has been associated with a substantial economic burden in a country-level cost analysis [6].

At present, standard therapies for retinal angiogenic disease, including DR and nAMD, rely predominantly on intravitreal anti-vascular endothelial growth factor (anti-VEGF) agents, with adjunctive corticosteroids and laser photocoagulation in selected settings [7]. Although these interventions can improve macular edema and suppress neovascular activity, their pharmacodynamic duration necessitates repeated administration, often at monthly or near monthly intervals, thereby imposing substantial cumulative treatment burden. In addition, intravitreal therapy carries procedure related risks, including endophthalmitis, intraocular pressure elevation, and lens opacification in susceptible eyes [8]. A substantial subset of patients exhibit suboptimal anatomical or functional responses despite intensive anti-VEGF regimens, underscoring the unmet need for more durable, disease-modifying strategies that target the upstream biology of angiogenesis and vascular barrier dysfunction [9].

Gene therapy can modify core molecular processes underlying DR, including VEGF overexpression, persistent inflammation, and oxidative stress, by adding or altering therapeutic genes in retinal cells. The most commonly used delivery vehicles are adeno-associated virus (AAV) vectors, which have a favorable safety record, low immunogenicity, and capacity for long-term transgene expression [10, 11].

Although early-phase trials and preclinical studies have shown promising outcomes, including reduced neovascularization and improved retinal architecture, clinical translation remains incomplete [12]. The key obstacles are heterogeneity in trial design, gene constructs, vector platforms, administration routes, and long-term safety and efficacy data. Moreover, the lack of a broad synthesis of clinical evidence limits informed decision-making by clinicians, researchers, and health policymakers.

Despite strong biological rationale and accumulating early translational evidence, the gene therapy literature in retinal angiogenic disease remains fragmented across in vitro systems, animal models, and early human trials. Interpretation is further constrained by substantial heterogeneity in vector platforms, transgene constructs, delivery routes, follow-up durations, and outcome metrics, which collectively limits inference regarding the consistency, magnitude, and translatability of therapeutic effects. Consequently, an integrated synthesis that prioritizes suppression of pathological neovascularization, protection of the vascular barrier, and clinically relevant functional endpoints across translational stages remains lacking.

Therefore, this systematic review and meta-analysis aimed to evaluate the efficacy, safety, and translational relevance of ocular gene therapy targeting angiogenesis and vascular leakage across retinal vascular and neovascular disorders. The primary objective was to quantify the effect of gene-based interventions on pathological neovascularization and vascular leakage across experimental systems using standardized quantitative synthesis. The secondary objective was to synthesize anatomical and functional outcomes in human studies, explicitly recognizing that available trials span multiple retinal indications and primarily serve as proof of concept for ocular gene delivery and durability. The exploratory objective was to investigate sources of heterogeneity through subgroup analyses and meta-regression, focusing on vector class, transgene category, and pathway-level mechanism of action. Collectively, these objectives were designed to provide a comprehensive translational assessment and inform future clinical development of gene therapy for retinal vascular diseases.

## Methodology

### Protocol registration and reporting standards

This systematic review and meta-analysis were prospectively registered in the International Prospective Register of Systematic Reviews (PROSPERO; registration number CRD420251080239). The conduct and reporting of the review followed the Preferred Reporting Items for Systematic Reviews and Meta-Analyses (PRISMA 2020) guidelines.

### Search strategy

A comprehensive and systematic literature search was undertaken to identify relevant preclinical and clinical studies evaluating gene-based therapeutic strategies for retinal diseases, with particular emphasis on angiogenesis, neovascularization, and visual and anatomical outcomes. The electronic databases PubMed (MEDLINE), Scopus, Web of Science, ScienceDirect, and the Cochrane Library were initially searched from inception to December 21, 2025, and subsequently updated through April 21, 2026. The search strategy combined controlled vocabulary terms (e.g., Medical Subject Headings (MeSH) in PubMed) with free-text keywords, using Boolean operators (“AND”, “OR”) to optimize sensitivity and specificity. Search terms were structured around four core concepts: (1) gene therapy and viral vectors (e.g., adeno-associated virus, adenovirus, lentivirus), (2) retinal and chorioretinal diseases (e.g., diabetic retinopathy, age-related macular degeneration, choroidal neovascularization, inherited retinal dystrophies), (3) angiogenesis-related outcomes (e.g., neovascularization, vascular leakage, VEGF signaling), and (4) functional and structural endpoints (optical coherence tomography, central subfield thickness) as shown in Table 1. The complete database-specific search strings and Boolean combinations are provided in Table 1. Additional studies were identified through manual screening of reference lists from included articles and relevant reviews. All retrieved records were exported to reference management software, and duplicates were removed prior to screening. Study selection was independently performed by two reviewers, who screened titles and abstracts followed by full-text assessment against predefined eligibility criteria. Disagreements were resolved by consensus.

**Table 1:** Search strategy

Database	Search string (Boolean Strategy)
PubMed (MEDLINE)	(“Gene Therapy”(Mesh) OR “gene therapy” OR “viral vector*” OR AAV OR adenovirus OR lentivirus) AND (“Retinal Diseases”(Mesh) OR retina* OR “diabetic retinopathy” OR “age-related macular degeneration” OR AMD OR “choroidal neovascularization” OR CNV) AND (angiogenesis OR neovascularization OR VEGF OR sFLT-1 OR PEDF OR endostatin OR angiostatin) AND (OCT OR “central subfield thickness” OR CST)
Scopus	TITLE-ABS-KEY ( “gene therapy” OR “viral vector*” OR AAV OR adenovirus OR lentivirus) AND TITLE-ABS-KEY ( retina* OR “diabetic retinopathy” OR “macular degeneration” OR AMD OR “choroidal neovascularization”) AND TITLE-ABS-KEY ( angiogenesis OR neovascularization OR VEGF OR sFLT-1 OR PEDF OR endostatin OR angiostatin) AND TITLE-ABS-KEY ( OCT OR “central subfield thickness”)
ScienceDirect	(“gene therapy” OR “AAV” OR “viral vector” OR adenovirus OR lentivirus) AND (retina OR “diabetic retinopathy” OR AMD OR “choroidal neovascularization”) AND (angiogenesis OR neovascularization OR VEGF OR sFLT-1 OR PEDF) AND (OCT OR BCVA)
Cochrane Library	(gene therapy OR viral vector OR AAV):ti,ab,kw AND (retina OR diabetic retinopathy OR macular degeneration OR AMD):ti,ab,kw AND (angiogenesis OR neovascularization OR VEGF OR sFLT-1):ti,ab,kw
Web of science	TS = (“gene therapy” OR “viral vector*” OR AAV OR adenovirus OR lentivirus) AND TS = (retina* OR “diabetic retinopathy” OR “macular degeneration” OR AMD OR “choroidal neovascularization”) AND TS = (angiogenesis OR neovascularization OR VEGF OR sFLT-1 OR PEDF OR endostatin OR angiostatin) AND TS = (OCT OR “central subfield thickness”)

### Study selection and eligibility

Study selection was conducted in accordance with the Population, Intervention, Comparator, Outcome (PICO)framework to ensure methodological clarity and clinical relevance. The population (P) comprised human participants with retinal diseases characterized by angiogenesis or vascular dysfunction, including neovascular age-related macular degeneration and diabetic retinopathy, together with non-angiogenic inherited or mitochondrial retinal disorders included only for translational functional-outcome contextualization, as well as relevant in vitro and animal models of retinal neovascularization. The intervention (I) included gene-based therapies delivered using viral vectors, particularly adeno-associated virus (AAV), adenoviral, or lentiviral systems targeting angiogenic, anti-angiogenic, antioxidant, or retinal functional pathways. The comparators (C) consisted of untreated controls, sham interventions, standard of care, or baseline pre-treatment measurements. The outcomes (O) of interest included measures of angiogenesis or vascular leakage (e.g., neovascular area or preretinal neovascular nuclei counts, fluorescein leakage scores or endothelial permeability assays), structural retinal outcomes, including optical coherence tomography (OCT)-derived central subfield thickness (CST), and functional endpoints such as best-corrected visual acuity (BCVA).

Outcome-domain eligibility was predefined to ensure consistency across heterogeneous indications and translational stages. Studies were considered eligible if they reported at least one of the prespecified outcome domains: (1) neovascularization indices (e.g., neovascular area, preretinal neovascular nuclei counts, choroidal neovascularization lesion metrics), (2) vascular permeability or leakage indices (e.g., fluorescein leakage scores, endothelial permeability assays, blood retinal barrier integrity outcomes), (3) functional outcomes, primarily best-corrected visual acuity (BCVA) reported in logMAR or in formats convertible to logMAR, (4) structural outcomes, primarily central subfield thickness (CST), or (5) treatment-burden outcomes, primarily supplemental anti-VEGF injection frequency. Indication heterogeneity was anticipated, and therefore quantitative pooling was performed within outcome domains rather than within a single disease indication, with results interpreted in the context of translational stage and construct class.

Eligible studies included randomized controlled trials, non-randomized clinical studies, preclinical in vivo experiments, and in vitro mechanistic studies reporting relevant outcomes. Exclusion criteria comprised non-gene-based interventions, reviews, editorials, case reports, conference abstracts without full data, studies lacking relevant outcomes, articles published before the year 2000, and articles not published in English. In addition, human observational studies without gene transfer interventions were included only for translational contextualization of disease mechanisms or outcome interpretability, and were not eligible for quantitative pooling. Titles and abstracts were screened independently by two reviewers, followed by full-text assessment. Discrepancies were resolved through discussion and consensus, ensuring transparent and reproducible study selection.

### Quality assessment and critical appraisal

The methodological quality and risk of bias of the included studies were systematically assessed using validated appraisal tools appropriate to study design. Randomized controlled trials were evaluated using the Cochrane Risk of Bias 2 (ROB 2) tool, which assesses bias arising from the randomization process, deviations from intended interventions, missing outcome data, outcome measurement, and selective reporting. Non-randomized and observational human studies were appraised using the Risk Of Bias In Non-randomized Studies of Interventions (ROBINS-I) tool, focusing on bias due to confounding, participant selection, intervention classification, deviations from intended interventions, missing data, outcome measurement, and reporting bias. Preclinical animal studies were assessed using the SYRCLE risk of bias tool, which is specifically designed for animal intervention studies and evaluates domains such as sequence generation, allocation concealment, blinding, housing conditions, and outcome reporting. Quality assessment was conducted independently by two reviewers, with disagreements resolved through discussion and consensus. No study was excluded solely on the basis of quality; instead, risk-of-bias assessments were used to inform interpretation of findings and to contextualize the strength of the evidence. Overall, clinical trials demonstrated low to moderate risk of bias, while observational studies showed moderate risk primarily due to confounding. Preclinical studies were generally rated as low to unclear risk, largely reflecting incomplete reporting rather than confirmed methodological flaws. This structured appraisal ensured transparent and robust evaluation of the evidence base.

### Data extraction and data synthesis

Data extraction was performed using a standardized, pre-piloted extraction form developed specifically for this review to ensure consistency and accuracy. For each eligible study, the following information was extracted: author and year of publication, study design and country, population characteristics or experimental model, sample size, type of gene therapy and vector used, route of delivery, comparator or control conditions, duration of follow-up, and outcomes of interest. Extracted outcomes included angiogenesis-related measures (e.g., neovascular area, vascular leakage), structural retinal parameters, including OCT-derived CST, functional outcomes including BCVA, and safety data where applicable. When quantitative data were not directly reported, values were estimated from figures using validated graphical extraction methods. Data extraction was independently conducted by two reviewers, and discrepancies were resolved through discussion and consensus. In cases of missing or unclear information, corresponding authors were contacted when feasible. This rigorous and systematic data extraction approach was designed to minimize errors and ensure the reliability of the synthesized evidence.

### Statistical analysis and narrative synthesis

Quantitative synthesis was performed where sufficient homogeneity in study design, intervention, and outcome reporting was present. Meta-analyses were conducted using Comprehensive Meta-Analysis (CMA) software. Random-effects models were used as the primary approach to account for expected clinical and methodological heterogeneity across translational stages, vector platforms, constructs, and outcome definitions. Fixed-effects models were additionally applied as sensitivity analyses when statistical heterogeneity was minimal, and estimates from both approaches were compared to assess robustness of pooled effects. Continuous outcomes were summarized using mean differences (MD) or standardized mean differences (SMD; Hedges’ g) with corresponding 95% confidence intervals (CIs), depending on consistency of outcome measurement scales. Statistical heterogeneity was assessed using the Cochran Q test and quantified with the I^2^ statistic, with I^2^ values of 25%, 50%, and 75% representing low, moderate, and high heterogeneity, respectively. Prespecified subgroup analyses were planned to explore heterogeneity across translational stage (in vitro, animal, and human studies) and by construct class/mechanistic category, including anti-VEGF pathway modulation [e.g., soluble fms-like tyrosine kinase-1 (sFLT-1), pigment epithelium-derived factor (PEDF)], antioxidant strategies [e.g., manganese superoxide dismutase (MnSOD)], and other non-VEGF angiogenesis-related constructs. Meta-regression was performed where sufficient studies were available to evaluate mechanistic pathway categories as moderators. For human studies, pooled clinical outcomes, including CST change and anti-VEGF treatment-burden reduction, were interpreted as proof-of-concept signals of ocular gene delivery, durability, and biological activity across retinal indications, rather than as diabetic retinopathy-specific efficacy unless diabetic retinopathy-dedicated clinical trials were available and analyzable. Where quantitative pooling was not appropriate due to heterogeneity, limited data, or differences in outcome reporting, findings were synthesized using a structured narrative approach. Narrative synthesis was conducted by grouping studies according to experimental model (in-vitro, animal, human), therapeutic strategy, and outcome domain (angiogenesis, vascular leakage, structural and functional measures). Direction and magnitude of effects were summarized alongside methodological quality and risk-of-bias assessments to contextualize the strength of evidence. Together, the combined quantitative and narrative synthesis approaches provided a comprehensive evaluation of the translational impact of gene therapy across preclinical and clinical settings.

Studies were categorized into two conceptual groups to ensure biologically appropriate interpretation:

1. Mechanism-validation studies, including preclinical models and angiogenesis-driven clinical conditions (e.g., neovascular AMD and diabetic retinopathy).
2. Translational functional studies involving non-angiogenic retinal disorders [e.g., Leber hereditary optic neuropathy (LHON), retinal pigment epithelium-specific 65 kDa protein-Leber congenital amaurosis (RPE65-LCA), and X-linked retinitis pigmentosa (XLRP)].

Outcomes such as neovascularization and vascular leakage were interpreted as direct measures of anti-angiogenic efficacy, whereas CST outcomes from non-angiogenic conditions were interpreted as indicators of gene therapy feasibility, transgene expression, and functional restoration rather than disease-specific anti-angiogenic effects. Preclinical studies included in the neovascularization meta-analysis were stratified based on model type into oxygen-induced retinopathy (OIR), CNV, and VEGF-driven or metabolic angiogenesis models. This subgrouping was implemented to account for differences in anatomical location, disease induction, and vascular compartments. Despite these differences, all models share a common VEGF-mediated angiogenic pathway, allowing pathway-level synthesis. Standardized mean differences (SMDs) were used to enable pooling of outcomes measured on different scales.

## Results

**Fig. 1:**
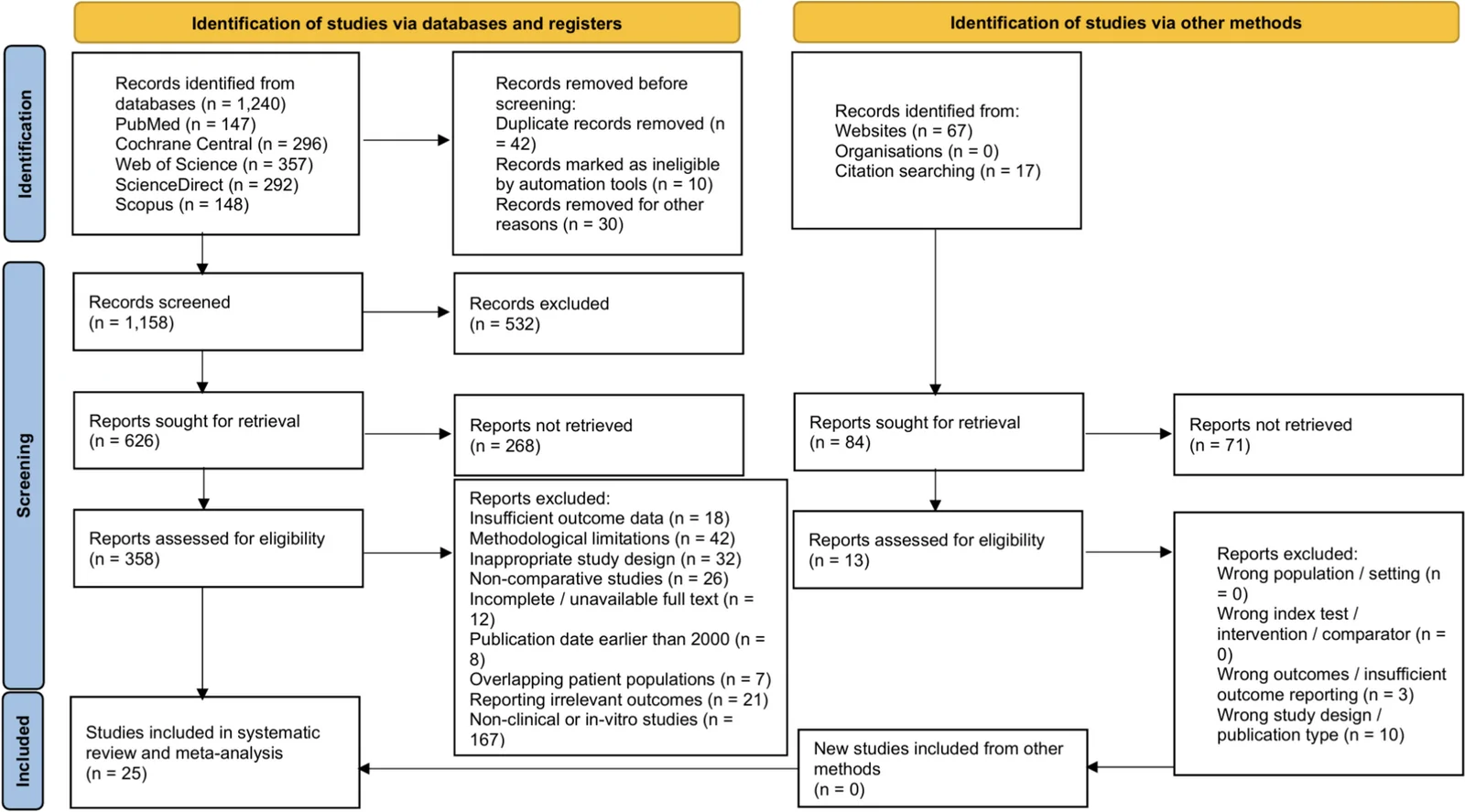
Preferred Reporting Items for Systematic Reviews and Meta-Analyses (PRISMA) 2020 flow diagram summarizing study identification, screening, eligibility assessment, and included studies

This PRISMA 2020 flow diagram summarizes the systematic study selection process from both database searching and other methods. In the identification stage via databases and registers, 1,240 articles were identified, including 147 articles from PubMed, 296 articles from Cochrane Central, 357 articles from Web of Science, 292 articles from ScienceDirect, and 148 articles from Scopus. Before screening, 42 duplicate articles were removed, 10 articles were marked as ineligible by automation tools, and 30 articles were removed for other reasons, leaving 1,158 articles for title and abstract screening. During the screening stage, 532 articles were excluded because they did not meet the preliminary relevance criteria, and therefore 626 articles were sought for retrieval. Among these, 268 articles could not be retrieved, resulting in 358 full-text articles assessed for eligibility. After full-text evaluation, 333 articles were excluded for predefined reasons, including 18 articles with insufficient outcome data, 42 articles not meeting prespecified methodological eligibility criteria, 32 articles with inappropriate study design, 26 non-comparative articles, 12 articles with incomplete or unavailable full text, 8 articles published earlier than 2000, 7 articles with overlapping patient populations, 21 articles reporting irrelevant outcomes, and 167 non-clinical or in vitro articles not meeting the prespecified eligibility criteria. In parallel, 84 articles were identified through other methods, including 67 articles from websites, 0 articles from organisations, and 17 articles from citation searching. All 84 articles were sought for retrieval, of which 71 articles were not retrieved, leaving 13 articles assessed for eligibility. These 13 articles were all excluded after eligibility assessment, including 0 articles excluded for wrong population or setting, 0 articles excluded for wrong index test, intervention, or comparator, 3 articles excluded for wrong outcomes or insufficient outcome reporting, and 10 articles excluded for wrong study design or publication type. Consequently, no new articles were included from other methods, and 25 articles from the database search met all eligibility criteria and were included in the systematic review; eligible interventional studies contributed to prespecified quantitative meta-analyses (Fig. 1).

### Study characteristics

The included studies spanned retinal angiogenic and neurovascular disorders, enabling outcome-domain based synthesis across translational stages while acknowledging heterogeneous clinical indications. Despite differences in disease indications, the studies converged on shared mechanistic pathways involving pathological angiogenesis and vascular barrier dysfunction, supporting cross-indication synthesis within prespecified outcome domains (Table 2). For methodological clarity, included records were stratified into interventional gene transfer studies and contextual observational studies. Interventional gene transfer studies comprised preclinical in vivo or in vitro vector mediated interventions and human trials administering gene transfer, and these studies were eligible for quantitative pooling within prespecified outcome domains. Contextual observational studies without gene transfer interventions were retained only to support translational interpretation of disease mechanisms, baseline phenotyping, and outcome contextualization, and were summarized narratively without inclusion in any meta-analysis. Clinical evidence comprised early to late phase trials, including phase I open-label studies (Heier et al., Feuer et al.), phase IIa randomized trials (Constable et al.), and a phase III randomized controlled trial (Newman et al. [28]), predominantly conducted in the USA and Australia. These studies evaluated intravitreal or subretinal AAV-based gene therapies targeting VEGF pathway modulation (e.g., sFLT-1) or retinal/mitochondrial targets [e.g., NADH dehydrogenase subunit 4 (ND4), retinal pigment epithelium-specific 65 kDa protein (RPE65), and retinitis pigmentosa GTPase regulator (RPGR)], and primarily contributed safety, durability, and functional outcome data [best-corrected visual acuity (BCVA)] alongside structural endpoints assessed by optical coherence tomography (OCT). Preclinical in vivo studies formed a substantial proportion, employing mouse, rat, and non-human primate models of ischemic retinopathy, choroidal neovascularization, and diabetic retinopathy, and predominantly used AAV, adenoviral, or lentiviral vectors delivering anti-angiogenic constructs (sFLT-1, PEDF, endostatin, angiostatin) or antioxidant strategies (MnSOD). Outcomes included neovascularization indices, vascular permeability or blood–retinal barrier integrity measures, and histological endpoints, with mechanistic biomarkers reported in selected studies. Complementary in vitro and computational studies explored angiogenic mechanisms and endothelial barrier function. Overall, the included studies demonstrate methodological and biological diversity across translational stages, while sharing core pathogenic pathways relevant to the prespecified outcome domains. It should be noted that only a subset of included studies evaluated angiogenesis-driven retinal diseases, while others involved inherited or mitochondrial retinal disorders such as LHON, RPE65-associated LCA, and XLRP. These conditions are not mediated by VEGF-driven angiogenesis or vascular leakage.

**Table 2:** Characteristics and main findings of the studies included

Study ID (Author, Year)	Study design, country	Population / model	Delivery method	Outcomes measured	Key findings / Key points
Heier et al., [12]	Phase 1 open-label dose-escalation clinical trial, USA	19 patients with advanced neovascular AMD	Single intravitreal AAV2-sFLT01 injection	Safety, BCVA, OCT thickness, aqueous sFLT-1	Therapy was generally safe with variable transgene expression and heterogeneous anatomical and visual responses
Constable et al., [13]	Phase 2a randomized controlled trial, Australia	32 patients with wet AMD	Single subretinal recombinant adeno-associated virus-soluble fms-like tyrosine kinase-1 (rAAV-sFLT-1) plus pro re nata (PRN) ranibizumab	Safety, BCVA, OCT, rescue injections	Subretinal gene therapy was well tolerated and associated with reduced anti-VEGF retreatment need
Lam et al., [14]	Prospective observational clinical study, USA	LHON patients and mutation carriers	Non-interventional functional testing	VA, RNFL thickness, PERG	PERG detected early RGC dysfunction even in asymptomatic carriers
Haurigot et al., [15]	Preclinical in vivo gene therapy, Spain	TgIGF-I mice	Intravitreal AAV2-PEDF	Neovascularization, VEGF levels	Sustained PEDF expression markedly inhibited retinal neovascularization
Pechan et al., [16]	Preclinical in vivo gene therapy, USA	Mouse OIR model	Intravitreal AAV2-sFLT01	Retinal neovascularization, toxicity	Long-term anti-VEGF expression reduced pathological angiogenesis
Lai et al., [17]	Preclinical translational study, Australia/Singapore	VEGF-transgenic mice; non-human primates	Subretinal AAV2-sFLT-1	CNV regression, ERG, histology	Sustained VEGF inhibition preserved retinal structure and function
Lamartina et al., [18]	Preclinical in vivo gene therapy, Italy	Mouse and rat OIR models	Intravitreal HD-adenoviral sFLT-1	Neovascularization, transgene regulation	Inducible long-term anti-VEGF expression significantly reduced angiogenesis
Ideno et al., [19]	Preclinical in vivo gene therapy, Japan	Spontaneously diabetic Torii rats	Subretinal rAAV5-sFLT-1	FA changes, DR progression	Long-term sFLT-1 expression suppressed DR progression without systemic effects
Le Gat et al., [20]	Preclinical in vivo gene therapy, France/USA	Neonatal mouse OIR model	Intravitreal adenoviral endostatin/ATF	Neovascular nuclei counts	Anti-angiogenic gene transfer reduced neovascularization by ~ 80%
Igarashi et al., [21]	Preclinical in vivo gene therapy, Japan	Mouse ischemic retinopathy model	Intravitreal lentiviral angiostatin	VEGF levels, neovascularization	Stable angiostatin expression significantly inhibited pathological angiogenesis
Gehlbach et al., [22]	Preclinical in vivo gene therapy, USA	Mouse CNV and ischemic retinopathy models	Intravitreous/periocular adenoviral sFLT-1	CNV area, BRB leakage	Route-dependent VEGF inhibition reduced CNV and vascular permeability
Auricchio et al., [23]	Preclinical in vivo gene therapy, USA	Mouse ischemic retinopathy	Subretinal AAV-PEDF/endostatin/TIMP3	Retinal neovascularization	PEDF and endostatin showed strongest anti-angiogenic effects
Bainbridge et al., [24]	Preclinical in vivo gene therapy, UK	Mouse ischemic retinopathy	Intravitreal Ad/AAV-sFLT-1	Neovascularization, safety	VEGF blockade reduced pathological angiogenesis without retinal toxicity
Zhang et al., [25]	Preclinical in vivo gene therapy, China/USA	Diabetic rats with metabolic memory	Intravitreal AAV-MnSOD	Capillary loss, apoptosis, BM thickening	Antioxidant gene therapy attenuated DR and metabolic memory
Feuer et al., [26]	Phase I clinical trial, USA	LHON patients (n = 5)	Intravitreal scAAV2-ND4	BCVA, RNFL, safety	Demonstrated safety and early visual improvements in some patients
Jacobson et al., [27]	Phase I/II clinical trial, USA	RPE65-LCA patients (n = 15)	Subretinal AAV2-RPE65	BCVA, OCT, FST	Sustained functional improvement with acceptable safety
Newman et al., [28]	Phase III RCT, multicenter	LHON patients (n = 38)	Intravitreal rAAV2-ND4	BCVA (logMAR), RNFL	No significant inter-eye difference; bilateral improvement observed
Lam et al., [29]	Phase 2/3 randomized dose-expansion trial; multicenter (international); Ophthalmology 2024	Male patients ≥10 years with retinitis pigmentosa GTPase regulator (RPGR)-associated X-linked retinitis pigmentosa (XLRP); n = 29 (low dose, n = 10; high dose, n = 10; control, n = 9)	Subretinal injection of cotoretigene toliparvovec (BIIB112/AAV8-RPGR); low dose, 5 × 10¹⁰ vector genomes(vg)/eye; high dose, 2.5 × 10¹¹ vg/eye; untreated controls	Primary: percentage meeting microperimetry responder criteria (≥7-dB improvement at ≥5/16 central loci).Secondary: change from baseline in retinal sensitivity (central 16 loci; full 68 loci), low-luminance visual acuity (LLVA) change, proportions achieving ≥15-Early Treatment Diabetic Retinopathy Study (ETDRS) and ≥10-ETDRS letter LLVA gain; safety/serious adverse events (SAEs)	Primary endpoint was not met. The low-dose group showed significant improvement versus control in mean microperimetry sensitivity (P = 0.035) and LLVA at 12 months (P = 0.0498). No significant benefit was observed for high dose versus control. Fewer ocular SAEs occurred with low dose (3) than high dose (7). The trial was underpowered because of coronavirus disease 2019 (COVID-19)-related early termination.
Rakoczy et al., [30]	Phase I RCT, Australia	nAMD patients with active CNV	Subretinal rAAV.sFLT-1	Safety, BCVA, injection burden	Good safety profile; reduced need for rescue injections; stable vision
Constable et al., [31]	Phase I dose-escalation, Australia	Advanced nAMD patients	Subretinal rAAV.sFLT-1	Safety, BCVA, CPT, injection frequency	Long-term safety maintained; stabilization of disease; reduced injections
Campochiaro et al., [32]	Phase I, open-label, USA	Advanced nAMD patients	Subretinal lentiviral (RetinoStat)	Safety, angiographic leakage, transgene expression	Sustained expression; safe vector; limited efficacy in late-stage disease
Rakoczy et al., [33]	Phase I/IIa, randomized, Australia	nAMD patients	Subretinal rAAV.sFLT-1	Safety, BCVA, CST, injection burden	Safe with modest biological signal; reduced injections in some subgroups
Khanani et al., [34]	Phase I, multicentre, USA	nAMD patients responsive to anti-VEGF	Intravitreal ADVM-022 (AAV-aflibercept)	Safety, BCVA, CST, injection burden	Significant reduction in injections (80–98%); stable vision and CST
Campochiaro et al., [35]	Phase I/IIa, dose-escalation, USA	Previously treated nAMD patients	Subretinal RGX-314 (AAV8)	Safety, BCVA, CST, injection burden	Sustained anti-VEGF expression; improved anatomical outcomes; reduced injections
Sun et al., [36]	Prospective clinical study (early phase), China	nAMD patients	Intravitreal gene therapy (anti-VEGF vector)	CST, choroidal thickness, BCVA	Reduction in CST; improved structural outcomes; limited change in choroidal parameters

AAV, adeno-associated virus; BCVA, best-corrected visual acuity; BM, basement membrane; CNV, choroidal neovascularization; CPT, central point thickness; CST, central subfield thickness; COVID-19, coronavirus disease 2019; DR, diabetic retinopathy; ERG, electroretinography; ETDRS, Early Treatment Diabetic Retinopathy Study; FA, fluorescein angiography; FST, full-field stimulus testing; LLVA, low-luminance visual acuity; logMAR, logarithm of the minimum angle of resolution; nAMD, neovascular age-related macular degeneration; OCT, optical coherence tomography; OIR, oxygen-induced retinopathy; PEDF, pigment epithelium-derived factor; PERG, pattern electroretinography; PRN, pro re nata; RCT, randomized controlled trial; RGC, retinal ganglion cell; RNFL, retinal nerve fiber layer; SAE, serious adverse event; sFLT-1, soluble fms-like tyrosine kinase-1; VEGF, vascular endothelial growth factor; vg, vector genomes; XLRP, X-linked retinitis pigmentosa

### Risk of bias assessments

**Fig. 2:**
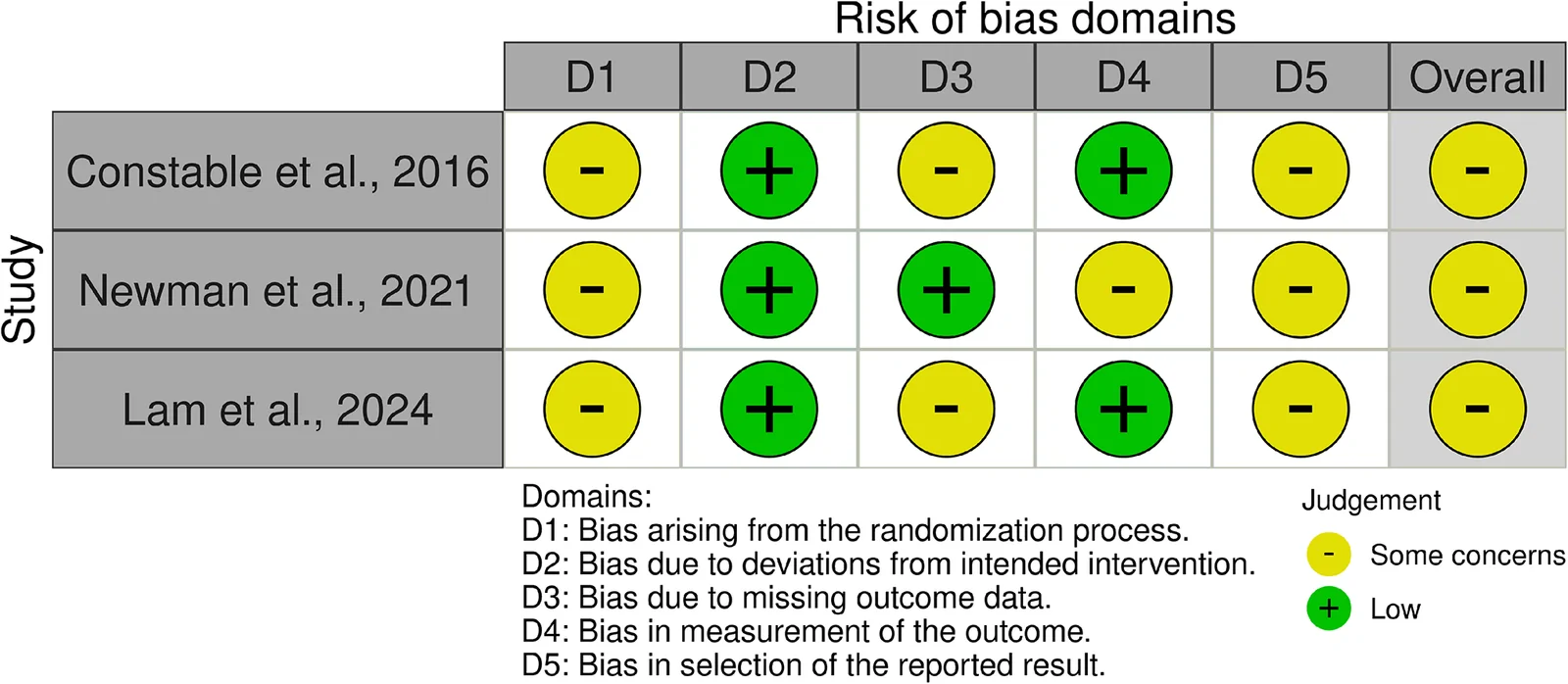
Risk of Bias 2 (ROB 2) traffic light plot summarizing risk of bias across domains for included randomized controlled trials

Risk of bias for the included randomized controlled trials was assessed using the ROB 2 tool, which evaluates bias across five domains related to trial conduct and reporting as shown in Fig. 2. Overall, trials Constable et al. [13], Newman et al. [28], and Lam et al. [29] were judged as having some concerns of bias, with no study rated at high risk in any domain. Bias arising from the randomization process (D1) was consistently rated as some concerns, reflecting limited reporting of allocation concealment and sequence generation, particularly in early-phase or underpowered trials. Deviations from intended interventions (D2) were judged as low risk across all studies, as treatment protocols were clearly defined and adherence was monitored. Missing outcome data (D3) raised some concerns in Constable et al. [13] and Lam et al. [29], the latter being affected by early termination related to the COVID-19 pandemic, whereas Newman et al. [28] demonstrated more complete follow-up. Measurement of outcomes (D4) was largely low risk due to objective assessments such as BCVA and OCT. Selection of reported results (D5) was rated as some concerns, reflecting multiple outcome analyses and post hoc explorations. Overall, the Risk of Bias 2 (ROB 2) assessment supports cautious interpretation while affirming acceptable methodological rigor for inclusion in quantitative synthesis (Fig. 2).

**Fig. 3:**
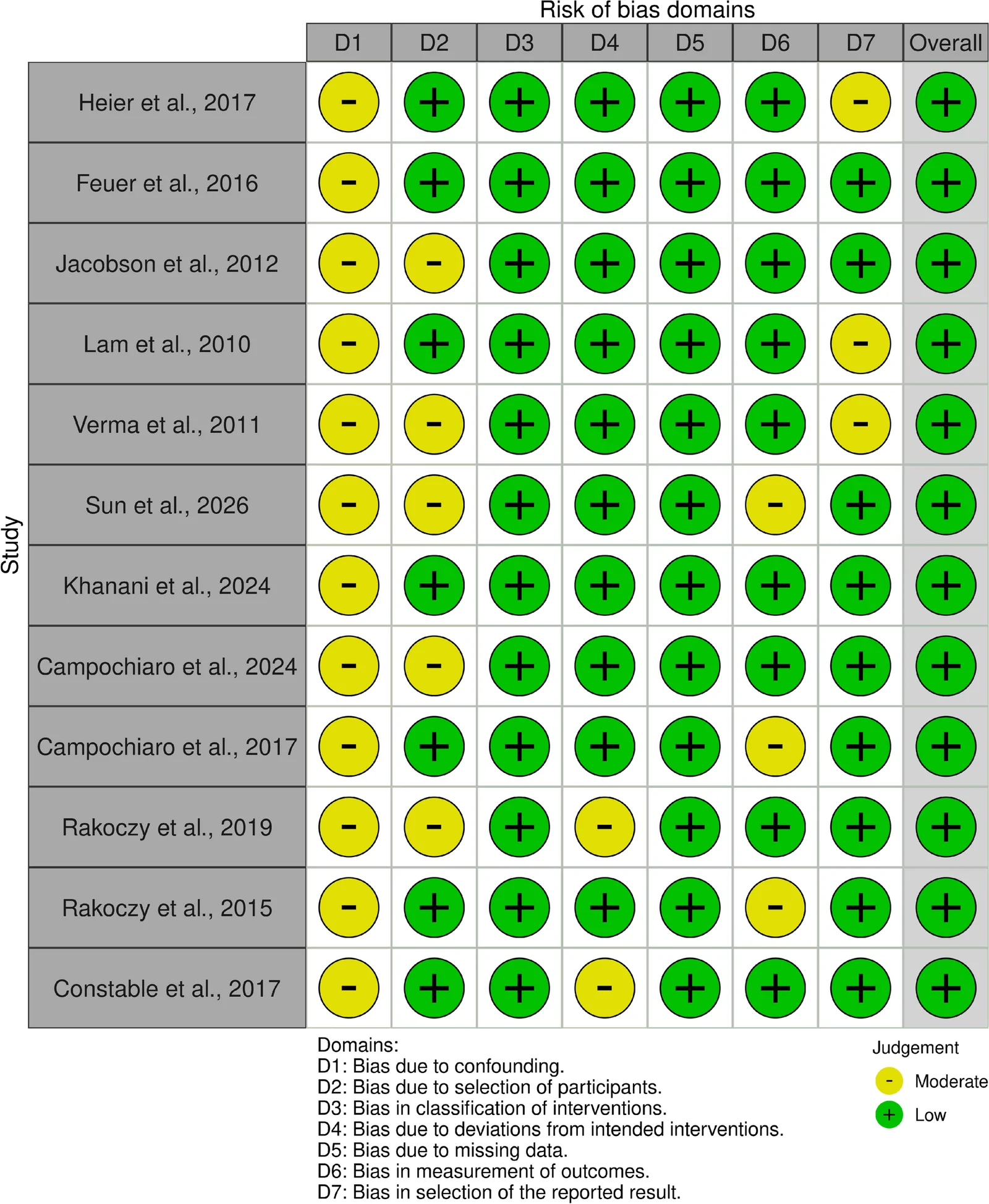
Risk Of Bias In Non-randomized Studies of Interventions (ROBINS-I) traffic light plot summarizing risk of bias across domains for included nonrandomized human studies

Risk of bias assessment of the included non-randomized studies, evaluated using the ROBINS-I tool, demonstrated an overall low to moderate risk of bias across domains, as illustrated in the traffic plot. The most common source of bias was confounding (D1), where the majority of studies were judged to have moderate risk due to their early-phase, open-label, and non-randomized designs, which inherently limit control over baseline differences and co-interventions. Similarly, selection bias (D2) showed moderate concerns in several studies, reflecting selective recruitment of patients with advanced or treatment-resistant disease, a common feature in phase I/II gene therapy trials. In contrast, bias in classification of interventions (D3) and bias due to missing data (D5) were consistently low across studies, as interventions were clearly defined (e.g., vector type, dose, delivery route) and follow-up was generally complete with minimal attrition. Bias due to deviations from intended interventions (D4) was low to moderate, with some studies lacking blinding but maintaining protocol adherence. Outcome measurement bias (D6) was predominantly low, supported by the use of objective endpoints such as central subfield thickness and anti-VEGF injection frequency. Selective reporting bias (D7) was also low overall, with most studies reporting prespecified outcomes. Despite moderate concerns in certain domains, particularly confounding and selection bias, the consistency of objective outcome measures and standardized reporting across studies supports the overall robustness and reliability of the evidence base. Verma et al. [39] appears in Fig. 3 for completeness of the descriptive methodological assessment; this study reports data from diabetic mice and rats and was not included among the twenty-five studies in the systematic review or in any quantitative synthesis. Lam et al. [14] also appears in Fig. 3; this study is a non-interventional functional testing and recruitment study, as indicated in Table 2, and does not itself evaluate a gene-transfer intervention.

**Fig. 4:**
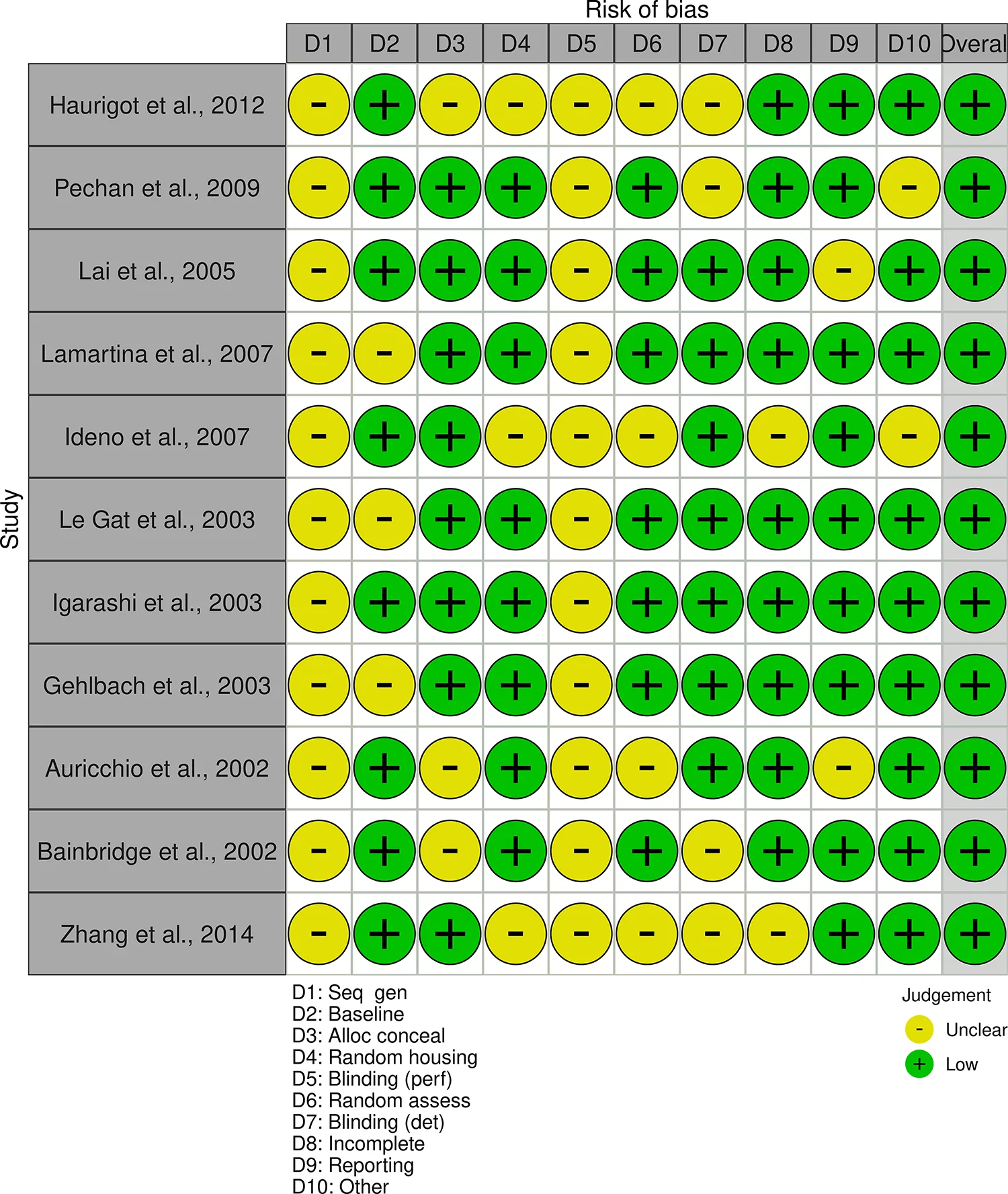
Systematic Review Centre for Laboratory Animal Experimentation (SYRCLE) risk-of-bias traffic light plot summarizing risk of bias across domains for included preclinical animal studies

Risk of bias for preclinical animal studies was assessed using the SYRCLE risk of bias tool, which is tailored to identify internal validity concerns specific to animal intervention experiments as shown in Fig. 4. Across the included studies, the overall risk of bias was judged as predominantly low to unclear, with no study demonstrating a consistently high risk across domains. Sequence generation (D1) and allocation concealment (D3) were frequently rated as unclear, as most studies including Auricchio et al. [23]; Bainbridge et al. [24]; and Le Gat et al. [20] did not explicitly describe randomization procedures or concealment methods. This likely reflects historical reporting practices rather than methodological omission, as these studies predate the ARRIVE guidelines. Haurigot et al. [15] also did not explicitly describe randomization or concealment methods; because this study was published in 2012, after the 2010 publication of the ARRIVE guidelines, this omission is reported here as an incomplete methodological description rather than as predating the ARRIVE guidelines. Baseline characteristics (D2) were generally rated as low risk, since disease models such as oxygen-induced retinopathy or diabetic rodents were consistently induced and compared with appropriate controls. Random housing (D4) and blinding of performance and outcome assessment (D5–D7) were often unclear, particularly in Pechan et al. [16]; Gehlbach et al. [22]; and Zhang et al. [25], where blinding was not explicitly stated. Nevertheless, outcome measures such as neovascular nuclei counts, CNV area, and capillary degeneration were objective and histologically defined, reducing the likelihood of detection bias. Incomplete outcome data (D8) and selective reporting (D9) were consistently assessed as low risk, as studies reported complete datasets without attrition or selective omission of outcomes.

### Angiogenesis

**Fig. 5:**
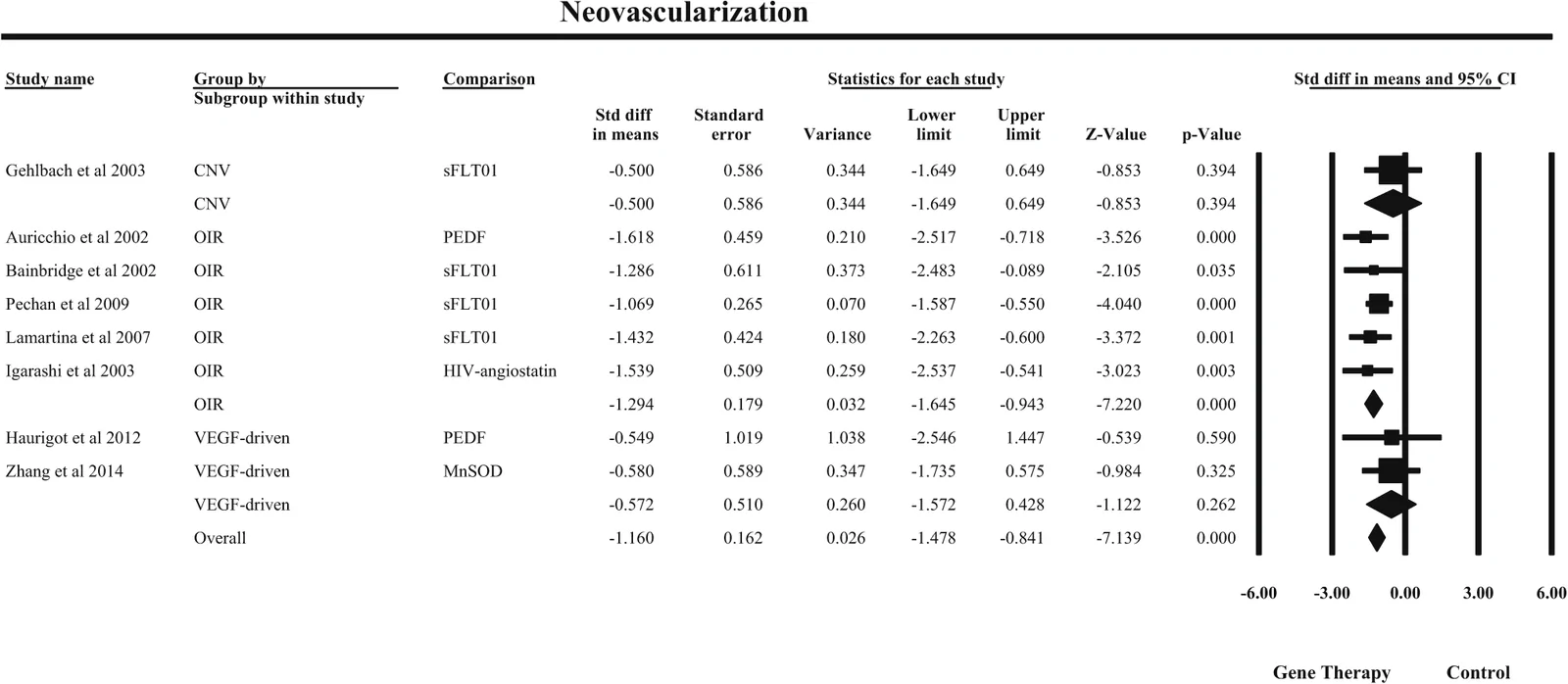
Forest plot of gene therapy-mediated inhibition of pathological neovascularization across preclinical models, stratified by model type and reported as standardized mean differences (SMDs). Negative SMD values favor gene therapy

Eight studies contributed to the neovascularization meta-analysis (Fig. 5). To address biological heterogeneity, preclinical models were stratified into OIR, CNV, and VEGF-driven or metabolic angiogenesis models. Gene therapy demonstrated consistent efficacy in suppressing pathological neovascularization across these subgroups, with a pooled standardized mean difference (SMD) of −1.16 (95% CI, −1.48 to −0.84; p < 0.001), indicating a reduction exceeding one standard deviation compared with controls and reflecting a strong anti-angiogenic effect. The OIR subgroup, including studies by Auricchio et al. [23], Bainbridge et al. [24], Pechan et al. [16], Lamartina et al. [18], and Igarashi et al. [21], demonstrated the most pronounced and consistent reduction (SMD ≈ −1.29; p < 0.001), highlighting robust suppression of ischemia-driven retinal angiogenesis. In contrast, CNV (Gehlbach et al. [22]) and VEGF-driven models (Haurigot et al. [15]; Zhang et al. [25]) showed smaller, non-significant effects, likely reflecting differences in vascular compartment, disease induction, and therapeutic responsiveness. Across studies, a range of gene therapy constructs—including AAV-, adenoviral-, and lentiviral-mediated delivery of sFLT-1, PEDF, angiostatin, and MnSOD—consistently targeted VEGF-related pathways. Despite differences in vector systems and dosing strategies, heterogeneity was negligible (I^2^ = 0%), supporting a shared underlying VEGF-mediated mechanism across models. The use of standardized mean differences enabled integration of diverse outcome measures, including neovascular area and endothelial nuclei counts. Clinically, these findings provide strong mechanistic evidence that ocular gene therapy can achieve potent and sustained suppression of pathological angiogenesis, supporting its translational potential in neovascular retinal diseases such as age-related macular degeneration and diabetic retinopathy.

### Vascular leakage

**Fig. 6:**
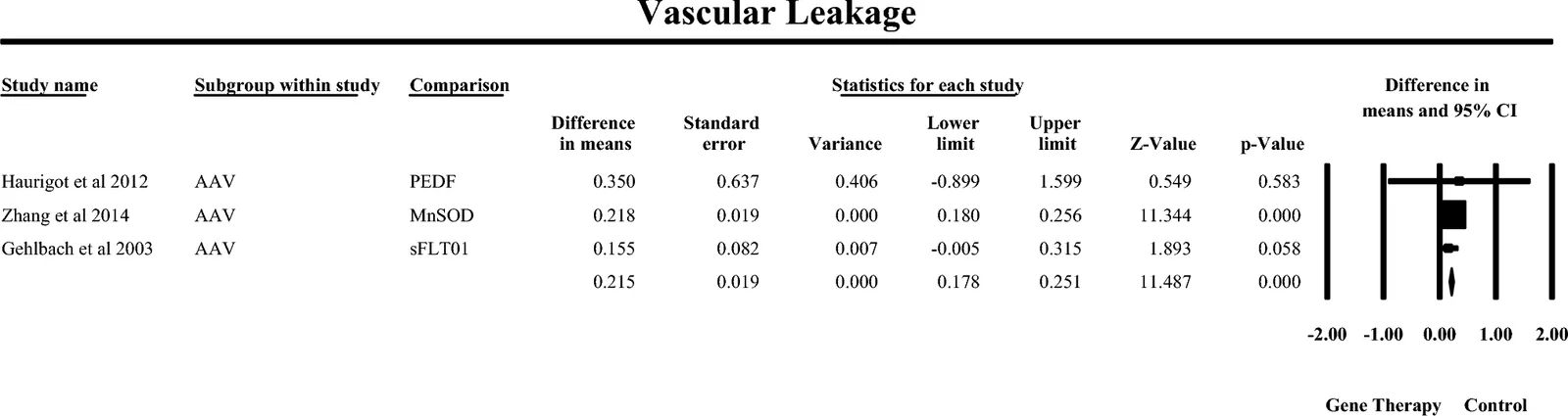
Forest plot of gene therapy effects on vascular leakage or permeability across preclinical studies, reported as directionally harmonized mean differences (MDs). Positive values indicate reduced leakage or permeability after gene therapy

Three preclinical experimental studies contributed to the vascular leakage meta-analysis (Fig. 6). Beyond this pooled analysis, additional preclinical evidence further supports gene therapy's protective effects on retinal structure and vascular integrity. Verma et al. [39] reported that adeno-associated virus 2 (AAV2)-mediated angiotensin-converting enzyme 2 (ACE2) or angiotensin-(1-7) [Ang-(1-7)] therapy in diabetic mice and rats reduced vascular permeability, acellular capillaries, oxidative stress, and inflammatory cell infiltration, restoring a vasoprotective balance in the retinal renin-angiotensin system. Similarly, Zhang et al. [25] showed that AAV2-MnSOD antioxidant gene therapy in diabetic rats prevented basement membrane thickening, decreased capillary apoptosis by >50%, and reduced acellular capillaries, indicating long-term mitigation of oxidative stress-induced retinal injury. These interventions collectively highlight the potential of gene therapy to preserve microvascular and neuronal integrity, attenuating DR progression beyond angiogenesis alone. Preclinical studies targeting VEGF pathways also reported structural benefits. Pechan et al. [16] and Lai et al. [17] observed preservation of photoreceptors and maintenance of retinal morphology in mice and non-human primates. Bainbridge et al. [24] confirmed normal retinal vascular development after sFlt-1 gene therapy, with transient procedural effects but no structural abnormalities, underscoring the safety of sustained VEGF inhibition.

In vitro endothelial barrier models demonstrated that sFLT-1 gene expression reduced VEGF-induced permeability by approximately 40 to 60%, accompanied by preservation of tight junction proteins and reduced transendothelial flux. Antioxidant gene transfer, including MnSOD-associated pathways, further reduced oxidative stress-induced barrier breakdown, with reductions in reactive oxygen species and endothelial apoptosis relative to controls. In vivo models supported translational consistency, with AAV- and adenovirus-mediated constructs reducing fluorescein leakage and vascular permeability indices. AAV- and adenovirus-mediated sFLT-1 and AAV-mediated PEDF delivery reduced fluorescein leakage scores by 30–70%, while antioxidantgene therapy reduced acellular capillaries by ~40–60%, indicating preservation of microvascular integrity.

Human trials demonstrated heterogeneous but quantifiable anatomical effects. In Heier et al., 2017, intravitreal AAV2-sFLT01 resulted in sustained reductions in CST in several participants, with individual eyes showing reductions up to  ~ 600 µm at week 18 and  ~ 860 µm by week 52, despite variability and intermittent rescue anti-VEGF use. Median center-point thickness changes demonstrated anatomical improvement in selected treated participants across follow-up. In Constable et al. [13], subretinal AAV therapy was associated with stabilization of central retinal thickness (≈300–380 μm range over 52 weeks) and maintenance of visual acuity, indicating anatomical safety and potential leakage control rather than progressive edema.

Figure 6 shows that three preclinical experimental studies assessing AAV- and adenovirus-mediated gene therapy demonstrated a significant reduction in vascular leakage after directional harmonization, with a pooled mean difference (MD) of 0.215 (95% CI, 0.178 to 0.251; p < 0.001). This MD indicates that AAV- and adenovirus-based gene transfer was associated with reduced vascular leakage on the directionally harmonized scale compared with control conditions. The contributing studies included Haurigot et al. [15] (AAV-PEDF), Zhang et al. [25] (AAV-MnSOD), and Gehlbach et al. [22] (Ad-sFLT01), representing complementary anti-angiogenic, antioxidant, and VEGF-neutralizing mechanisms. The forest plot in Fig. 6 groups the Gehlbach et al. [22] estimate under an AAV-labelled subgroup for display purposes; the original study used an adenoviral vector, consistent with the Ad-sFLT01 designation used here and in Table 2. Between-study heterogeneity was minimal (Q = 0.61, df = 2, p = 0.74; I^2^ = 0%), indicating a high degree of consistency across different transgenes and experimental systems. Identical pooled estimates under fixed- and random-effects models further support the robustness of the finding. Clinically, the observed reduction in endothelial permeability provides strong mechanistic support for downstream anatomical benefits observed in early human studies, including stabilization or reduction of macular thickness on OCT. Although derived from controlled preclinical experimental models, the magnitude and homogeneity of effect reinforce the translational relevance of viral vector-mediated gene therapy as a strategy to mitigate vascular leakage in retinal vascular diseases (Fig. 6).

**Fig. 7:**
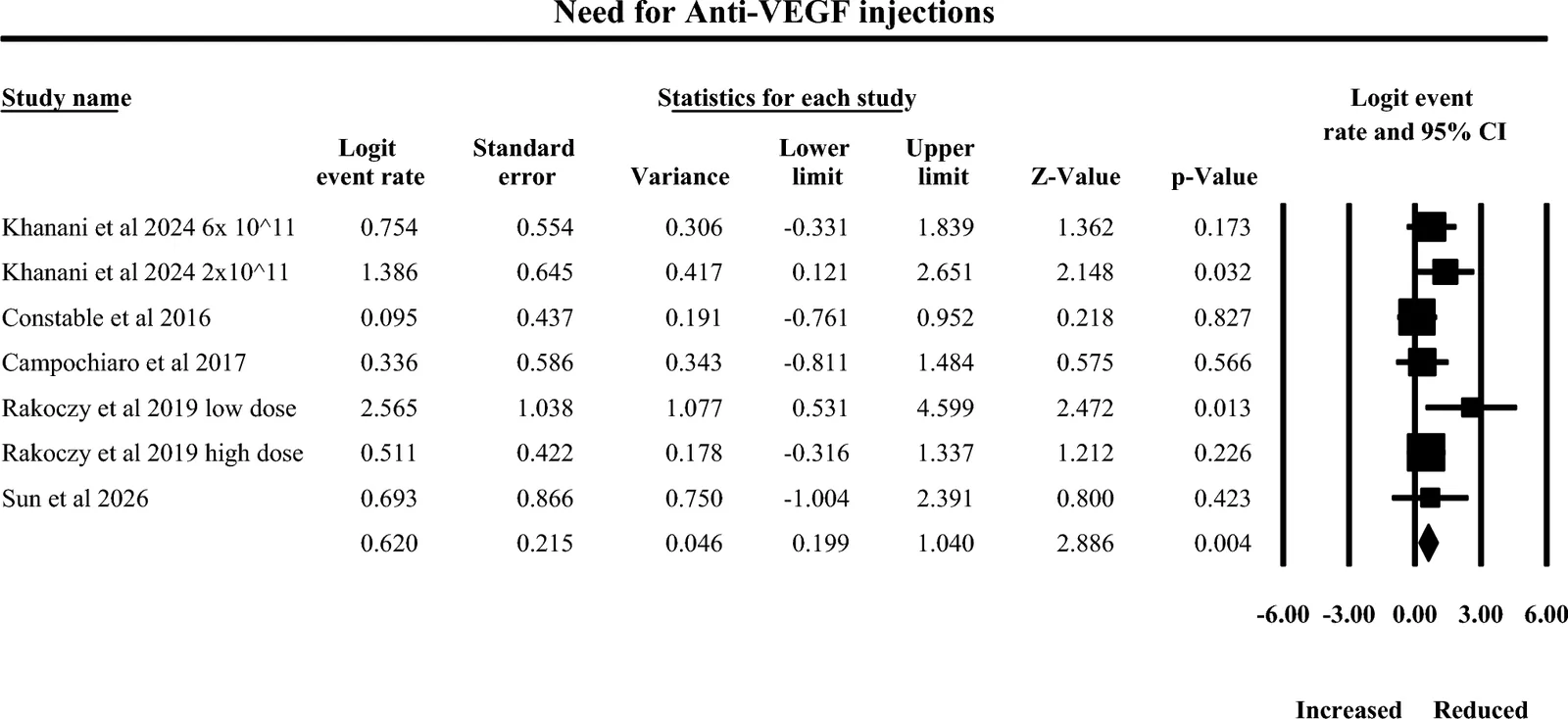
Forest plot of the pooled proportion of participants with reduced supplemental use of anti-vascular endothelial growth factor (anti-VEGF) after ocular gene therapy

### Anti-VEGF treatment burden

Quantitative synthesis of anti-VEGF treatment burden demonstrated a significant pooled proportion of participants meeting study-specific criteria for reduced supplemental anti-VEGF use following ocular gene therapy across seven clinical cohorts. Using a logit event rate model, the pooled estimate was 0.62 (95% CI 0.20 to 1.04; *p* = 0.0039), corresponding to a pooled proportion of approximately 65% of participants meeting study-specific criteria for reduced supplemental anti-VEGF use (inverse logit transformation). This indicates that, on average, nearly two-thirds of treated patients met study-specific criteria for a reduced need for supplemental anti-VEGF injections after gene therapy. Individual contributions were observed across multiple studies, including Khanani et al. [34], Constable et al. [13], Campochiaro et al. [32], Rakoczy et al. [33], and Sun et al. [36]. Effect sizes varied across cohorts, and the available data did not demonstrate a consistent monotonic dose-response gradient. Heterogeneity was low (I^2^ ≈ 11%, Q = 6.73, p = 0.35), indicating a consistent pooled pattern across the reported cohorts despite differences in vector platforms (AAV vs lentiviral), delivery routes, and dosing regimens. The agreement between fixed and random effects models further supports the directional consistency of the pooled cohort-level finding. Clinically, this result is highly relevant, as reduction in injection burden reflects sustained intraocular production of therapeutic anti-angiogenic proteins and decreased dependence on repeated intravitreal therapy. This outcome directly addresses one of the major limitations of current standard care—treatment burden and poor adherence—and highlights the potential of ocular gene therapy to provide durable, long-acting disease control in neovascular retinal disorders.

### Change in central subfield thickness

**Fig. 8:**
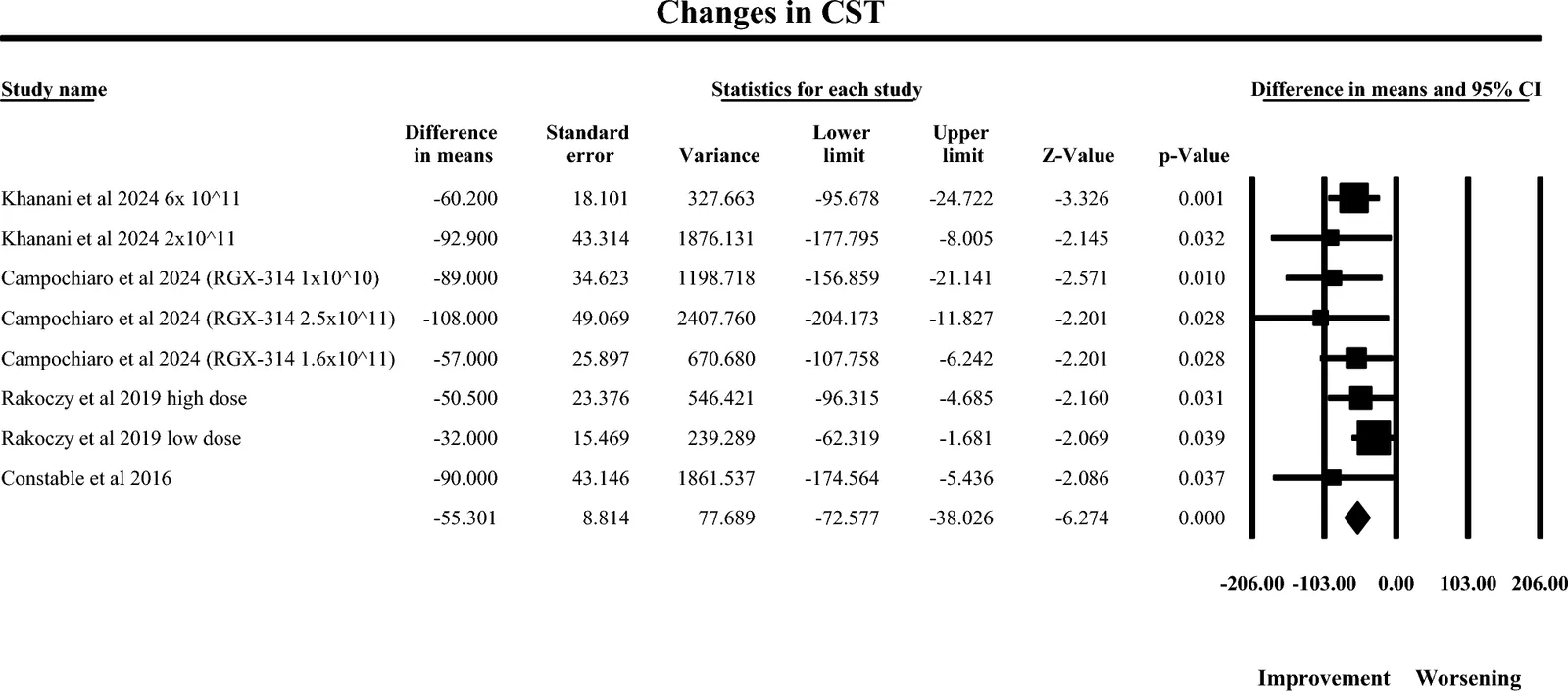
Forest plot of pooled changes in central subfield thickness (CST). Negative mean differences indicate anatomical improvement

Quantitative synthesis of CST (Fig. 8) demonstrated a statistically significant reduction following ocular gene therapy across eight clinical cohorts. The pooled MD of − 55.30 µm (95% CI − 72.58 to − 38.03; *p* < 0.001) indicates a consistent decrease in retinal thickness after treatment, reflecting improved control of retinal edema and vascular leakage. A negative MD in this context signifies anatomical improvement, as reduced CST corresponds to decreased intraretinal and subretinal fluid accumulation. Individual studies, including cohorts from Khanani et al. [34], Campochiaro et al. [35], Rakoczy et al. [33], and Constable et al. [13], all contributed to this effect, with reductions observed across a range of vector platforms and dosing regimens. CST reductions were observed across vector platforms and dose cohorts, without a consistent monotonic dose-response pattern. Heterogeneity was negligible (I^2^ = 0%, Q = 5.89, *p* = 0.55), indicating a high degree of consistency in treatment effect despite differences in study design, vector constructs, and dosing strategies. This supports the directional consistency of the pooled anatomical signal across the included dose cohorts and gene therapy approaches targeting VEGF pathways. Clinically, a reduction of approximately 55 µm in CST is meaningful, as it reflects improved retinal fluid homeostasis and reduced disease activity in neovascular retinal disorders. Importantly, CST serves as an objective structural biomarker bridging preclinical anti-permeability effects and clinical outcomes. These findings support the role of ocular gene therapy not only in mechanistic angiogenesis suppression but also in achieving sustained anatomical stabilization, which is critical for long-term disease control and reduction in treatment burden.

### Gene expression durability and mechanistic insights

Several studies evaluated the duration and localization of therapeutic gene expression, confirming stable, long-term activity of vector constructs. Haurigot et al. observed sustained PEDF expression for up to 18 months post-injection, with stable anti-angiogenic effects and no loss of vector efficacy. Igarashi et al. and Ideno et al. demonstrated localized retinal transgene expression persisting beyond 30 weeks, with no systemic dissemination.

Mechanistically, gene therapy was shown to modulate multiple molecular pathways implicated in diabetic retinopathy. Downregulation of VEGF, matrix metalloproteinase-2 and -9 (MMP2/9), connective tissue growth factor (CTGF), and inflammatory mediators was consistently observed, while upregulation of antioxidant enzymes, including manganese superoxide dismutase (MnSOD), contributed to endothelial stabilization and neuronal preservation.

**Fig. 9:**
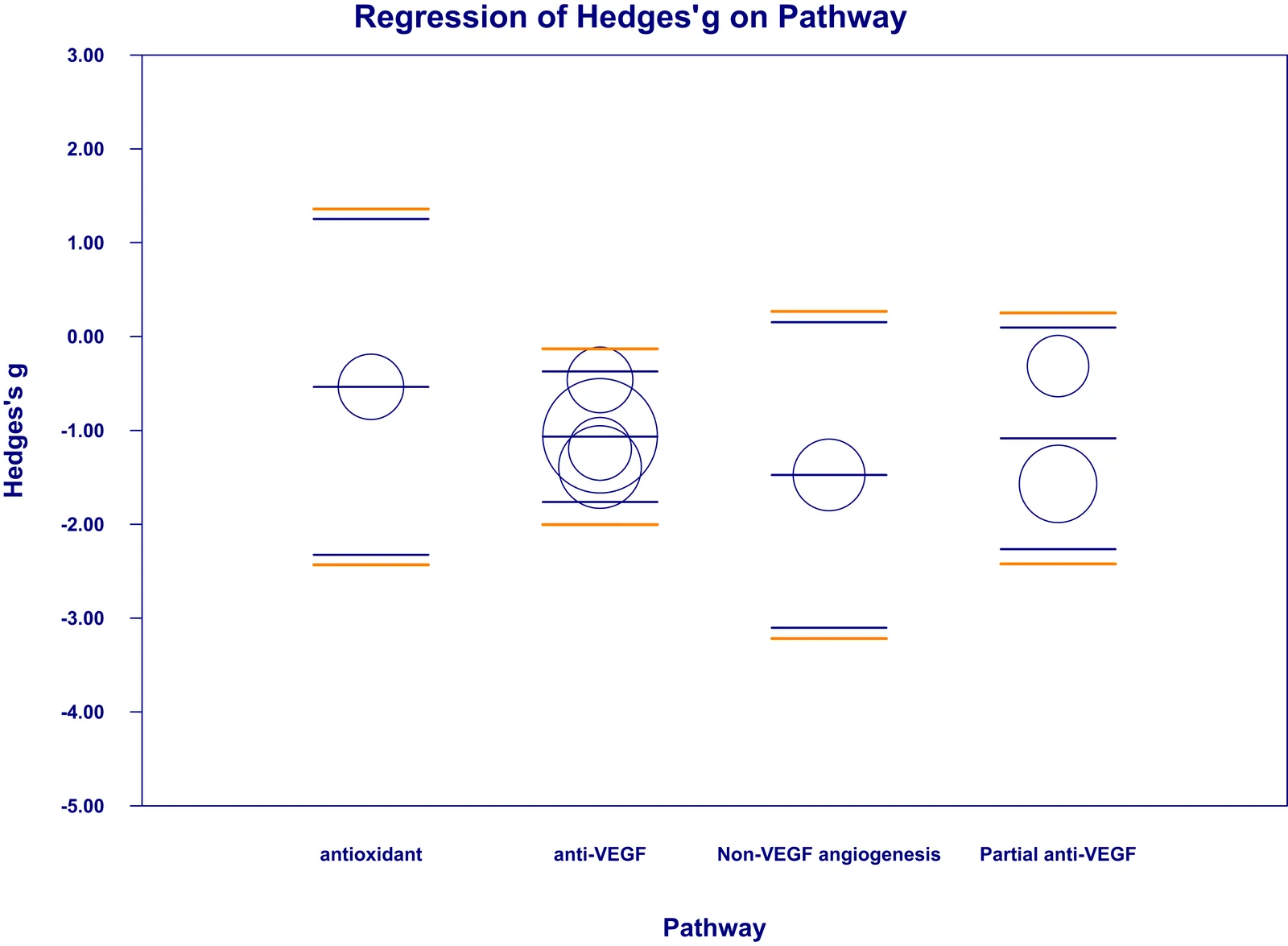
Meta-regression of neovascularization effect sizes by mechanistic pathway category across preclinical studies, expressed as Hedges' standardized mean difference (Hedges' g)

A random-effects meta-regression was performed to examine whether angiogenic pathway targeting explained variability in the inhibitory effect of gene therapy on neovascularization, expressed as Hedges’ g. Eight preclinical studies were included. Pathway classification (anti-VEGF, partial anti-VEGF, non-VEGF angiogenesis, and antioxidant) was entered as a categorical covariate. The overall model was not statistically significant (Q = 1.43, df = 3, p = 0.6977), indicating that no single pathway category independently accounted for between-study variability as shown in Fig. 9. The proportion of total between-study variance explained by the model was negligible (R^2^ analog = 0.00). Residual heterogeneity was low (τ^2^ = 0.0413; I^2^ = 17.65%; Q = 4.86, df = 4, p = 0.3022), suggesting relative consistency across studies once pathway grouping was considered. Despite the lack of statistical significance, directional effects were interpreted as exploratory signals. Anti-VEGF-based interventions demonstrated consistent negative coefficients (β = −0.5305; 95% CI, −1.7519 to 0.6909), indicating directionality toward reduced neovascularization within the coded pathway categories. Partial anti-VEGF approaches (e.g., PEDF-based strategies) showed a comparable directional coefficient (β = −0.5491), whereas non-VEGF angiogenesis inhibition and antioxidant pathways exhibited greater variability and less consistent magnitude of effect. Importantly, although no pathway emerged as statistically dominant, anti-VEGF signaling modulation showed the most coherent and biologically plausible pattern of angiogenesis inhibition across studies, aligning with established VEGF-driven mechanisms in retinal vascular disease. The absence of statistical significance is likely attributable to the small number of studies per subgroup and heterogeneity in outcome metrics (percentage vs area-based measures). Clinically, these findings reinforce anti-VEGF gene therapy as the most translationally relevant strategy for durable angiogenesis suppression, supporting its continued prioritization in retinal gene therapy development and providing mechanistic justification for observed visual and anatomical benefits in human trials.

### Publication bias

Publication bias was assessed across all outcomes using Begg and Mazumdar rank correlation, Egger’s regression intercept, and visual inspection of funnel plots (Supplementary Figs. 1–4). For neovascularization, both Begg’s test (Kendall’s tau = 0.32, *p* = 0.27) and Egger’s test (intercept = 0.92, *p* = 0.49) were non-significant, suggesting no strong evidence of publication bias; funnel plot symmetry further supported this observation. Similarly, for vascular leakage, no evidence of bias was detected (Begg’s tau = 0.00, *p* = 1.00; Egger’s intercept =  − 0.06, *p* = 0.71), although the small number of studies limits interpretability. In contrast, CST demonstrated potential small-study effects, with significant Begg’s test (tau =  − 0.68, *p* = 0.018) and Egger’s test (intercept =  − 2.03, *p* = 0.002), indicating possible asymmetry; however, trim-and-fill analysis suggested no missing studies, and the adjusted effect size remained unchanged, supporting robustness of the findings. For anti-VEGF injection burden, Begg’s test was non-significant (tau = 0.48, *p* = 0.13), but Egger’s test indicated potential bias (intercept = 2.69, *p* = 0.047). Trim-and-fill analysis imputed three missing studies, resulting in a reduced but still consistent effect estimate. Overall, while most outcomes showed minimal evidence of publication bias, caution is warranted for CST and injection burden outcomes, where statistical asymmetry may reflect small-study effects or underlying heterogeneity rather than true publication bias.

## Discussion

This systematic review and meta-analysis provides an outcome-domain based synthesis of ocular gene therapy evidence across translational stages, integrating mechanistic efficacy data from in vitro and in vivo models with functional signals derived from early human studies across retinal angiogenic and neurovascular indications. Rather than attributing pooled effects to a single disease entity, the present work frames gene-based interventions as targeting convergent biological pathways that underlie multiple retinal vascular and neovascular disorders, including pathological angiogenesis and vascular barrier dysfunction. Collectively, the findings support the potential of ocular gene therapy to produce durable pathway-level modulation, while highlighting important constraints in clinical translatability that should be explicitly acknowledged when interpreting pooled human outcomes.

First, mechanistic efficacy was strongest and most consistent for neovascularization endpoints in preclinical and in vitro experimental systems. Across these studies, gene therapy demonstrated a large anti-angiogenic effect, with pooled SMDs ranging from −1.29 to −1.16, indicating reductions in neovascularization exceeding one standard deviation compared with control conditions. Notably, within AAV-based studies alone, the pooled effect remained substantial (SMD = −1.06; p < 0.001) with negligible heterogeneity (I^2^ = 0%), underscoring the reproducibility of AAV-mediated approaches despite variation in experimental models and neovascularization metrics. These pooled results were supported by consistent directional effects across individual studies, including reductions in neovascular area or preretinal neovascular nuclei with VEGF-pathway modulation strategies such as sFLT-1, as well as anti-angiogenic factor delivery using PEDF and endostatin constructs. Although exploratory meta-regression did not reach statistical significance due to the limited number of studies and sparse subgroup representation, the observed directional patterns favored anti-VEGF pathway modulation, supporting its biological plausibility and translational prioritization in ocular gene therapy development.

Second, vascular barrier protection was supported by convergent mechanistic evidence, reinforcing gene therapy’s potential to address retinal vascular leakage as a complementary disease-relevant domain beyond angiogenesis suppression alone. Meta-analysis of vascular leakage outcomes from preclinical experimental studies demonstrated a significant directionally harmonized pooled mean difference (MD) of 0.215 (95% CI 0.178–0.251; p<0.001), with minimal heterogeneity, indicating consistent preservation of endothelial barrier integrity across mechanistically distinct constructs, including VEGF-neutralizing approaches (e.g., sFLT-1), anti-angiogenic factors (e.g., PEDF), and antioxidant strategies (e.g., MnSOD). Mechanistically, such effects are consistent with reduced VEGF-driven endothelial permeability, improved tight-junction stability, and attenuation of oxidative stress-associated endothelial injury. Importantly, while this pooled estimate is derived from controlled experimental systems, it provides biologically coherent support for the anatomical signals observed in early human studies, including reductions or stabilization of macular thickness metrics, suggesting that barrier function modulation may constitute a clinically meaningful component of gene therapy’s therapeutic profile in retinal vascular diseases.

These findings collectively highlight both the promise and the limitations of current translational evidence. While animal and in vitro models such as oxygen-induced retinopathy and choroidal neovascularization recapitulate key angiogenic phenotypes and barrier dysfunction, they do not fully reproduce the chronic systemic metabolic milieu, multi-layer neurovascular degeneration, and long-term inflammatory remodeling that characterize human diabetic retinopathy. This constitutes an important model validity limitation and contributes to translational uncertainty when extrapolating preclinical effect sizes into clinical disease modification. In addition, because human trial evidence remains limited in quantity and heterogeneous in design, the current synthesis provides stronger support for mechanistic pathway efficacy than for indication-specific clinical effectiveness. The translational relevance of the present findings is further supported by the emergence of several late-stage clinical gene therapy programs, including ABBV-RGX-314 [35, 37], ADVM-022 (ixo-vec) [34, 37], and 4D-150, which have advanced into phase II/III trials for neovascular retinal diseases. These next-generation therapies utilize optimized adeno-associated viral (AAV) vectors to enable sustained intraocular expression of anti-VEGF biologics following a single administration. Early clinical data from these programs demonstrate substantial reductions in anti-VEGF injection burden, with reported decreases of up to 80–97%, alongside stable visual and anatomical outcomes. Notably, 4D-150 incorporates dual inhibition of VEGF-A and VEGF-C, representing an evolution beyond earlier single-target approaches such as sFLT-1. Similarly, ADVM-022 offers an intravitreal delivery strategy, reducing the need for surgical intervention, while RGX-314 has demonstrated consistent durability across both subretinal and suprachoroidal delivery platforms. Importantly, the outcomes observed in these advanced clinical programs align closely with the findings of the present meta-analysis, particularly the significant reduction in central subfield thickness and anti-VEGF treatment burden. This concordance supports the biological validity of sustained intraocular VEGF suppression and reinforces the potential of gene therapy to transform current treatment paradigms from repetitive intravitreal injections to long-acting or single-administration therapies.

A key methodological consideration in this analysis is the distinction between biological mechanism and functional outcome interpretation. While angiogenesis and vascular leakage endpoints directly reflect vascular endothelial growth factor-mediated disease processes in retinal vascular disorders, some functional and structural outcomes were derived from heterogeneous clinical populations, including non-angiogenic conditions. These diseases involve distinct pathophysiological mechanisms, such as mitochondrial dysfunction or photoreceptor degeneration, rather than vascular proliferation. Therefore, improvements in visual function observed in these studies were interpreted as evidence of successful gene delivery and retinal functional modulation, rather than as direct anti-angiogenic therapeutic effects. This distinction ensures alignment between biological mechanism and clinical inference and avoids overgeneralization of efficacy across unrelated disease pathways. In the present study, the inclusion of additional recent clinical studies and the generation of updated forest plots for CST and anti-VEGF injection burden further strengthen the clinical relevance of the findings. These newer analyses capture emerging evidence from next-generation gene therapy approaches and demonstrate consistency with earlier strategies such as AAV-sFLT-1. Notably, the XLRP gene therapy study by Lam et al. [29] did not meet its primary endpoint, underscoring the importance of contextual interpretation of negative findings. Several factors explain this outcome, including suboptimal dose selection, advanced disease stage at the time of intervention, and heterogeneity in patient characteristics, which limit the capacity for functional recovery in degenerative retinal disorders. Additionally, external factors such as COVID-19–related disruptions, including premature trial termination and reduced follow-up, may have impacted outcome assessment and statistical power. Importantly, XLRP is a non-angiogenic condition characterized by photoreceptor degeneration rather than VEGF-mediated vascular pathology, and therefore therapeutic responses are not directly comparable to those observed in neovascular retinal diseases.

From a safety perspective, the available evidence suggests an acceptable early safety profile across translational stages, with adverse events in clinical settings largely reflecting procedure-related effects and transient ocular inflammation. Nevertheless, safety interpretation remains constrained by limited sample sizes, heterogeneous reporting standards, and incomplete long-term follow-up in early-phase studies. As ocular gene therapies advance toward broader clinical adoption, safety surveillance must remain focused on dose-related inflammatory risk, vector immunogenicity, and durability of transgene expression, particularly given the potential irreversibility of gene transfer in ocular tissues.

Some considerations should be noted when interpreting this synthesis. First, the included literature spans multiple translational stages and retinal indications, which is expected in an emerging ocular gene therapy field; importantly, we proactively minimized the impact of this heterogeneity by prespecifying outcome domains and conducting meta-analyses within these domains, rather than pooling across fundamentally different clinical entities. Second, human functional evidence remains less abundant than preclinical mechanistic data; clinical anatomical and treatment-burden outcomes were framed as proof-of-concept signals of ocular gene delivery, biological activity, and durability across indications rather than as definitive disease-specific efficacy. Third, for outcomes supported by a small number of studies, statistical tools for detecting small-study effects are inherently conservative; nevertheless, the consistency of effect direction, low observed heterogeneity in key analyses, and concordance between fixed- and random-effects estimates collectively support the robustness of the primary findings. Fourth, the forest plots in Fig. 7 and Fig. 8 include multiple dose cohorts from the same clinical trial, including the two Khanani et al. [34] dose groups, the three Campochiaro et al. [35] dose groups, and the two Rakoczy et al. [33] dose groups, entered as separate estimates; because dose cohorts from the same trial share a common study population and design, the pooled estimates are interpreted at the cohort level rather than as fully independent trial-level effects. Overall, these mitigation strategies enhance transparency and substantially improve the reliability of inference despite unavoidable differences in study design and outcome reporting.

Future research should prioritize adequately powered, disease-specific randomized clinical trials to validate anatomical and functional outcomes in clinically relevant retinal vascular populations, including diabetic retinopathy where systemic metabolic factors influence disease activity and therapeutic responsiveness. Standardization of outcome definitions for neovascularization and leakage endpoints, along with harmonized OCT-based structural metrics and consistent reporting of visual function, will improve comparability and enable more reliable quantitative synthesis. Long-term follow-up is essential to confirm durability of therapeutic expression, sustained efficacy, and late-onset adverse signals. In parallel, ongoing advances in vector engineering, regulated expression systems, and minimally invasive delivery approaches may further optimize the balance between efficacy, safety, and feasibility, ultimately supporting translation of ocular gene therapy from mechanistic promise into routine retinal clinical practice.

## Conclusion

This systematic review and meta-analysis demonstrates that ocular gene therapy provides strong preclinical anti-angiogenic and anti-permeability effects, with emerging clinical evidence of anatomical improvement and reduced anti-vascular endothelial growth factor (anti-VEGF) treatment burden in neovascular retinal disease cohorts. Preclinical evidence showed a large reduction in pathological neovascularization (standardized mean difference [SMD], −1.16; 95% confidence interval [CI], −1.48 to −0.84) and a significant decrease in vascular leakage after directional harmonization (MD, 0.215; p < 0.001). In clinical cohorts, gene therapy was associated with reduced central subfield thickness (CST; −55.3 μm; 95% CI, −72.6 to −38.0) and a pooled proportion of approximately 65% of participants meeting study-specific criteria for reduced anti-VEGF injection burden. Collectively, these findings support ocular gene therapy as a durable, long-acting strategy to reduce dependence on repeated intravitreal anti-VEGF injections and address treatment burden in neovascular retinal disorders. Larger disease-specific randomized trials remain essential to confirm long-term efficacy, safety, and patient selection.

## Supplementary Information

Below is the link to the electronic supplementary material.Supplementary file1 (PDF 116 KB)

## Data Availability

All data supporting the findings of this study are included within the article and its supplementary materials.

## Abbreviations

AAV: Adeno-associated virus
ACE2: Angiotensin-converting enzyme 2
ATF: Amino-terminal fragment (urokinase-type plasminogen activator related)
BCVA: Best-corrected visual acuity
BM: Basement membrane
BRB: Blood-retinal barrier
CNV: Choroidal neovascularization
CST: Central subfield thickness
CTGF: Connective tissue growth factor
DR: Diabetic retinopathy
ERG: Electroretinography
ETDRS: Early treatment diabetic retinopathy study
FA: Fluorescein angiography
FST: Full-field stimulus testing
HD-Ad: Helper-dependent adenovirus
Hedges’ g: Hedges’ standardized mean difference
HUVECs: Human umbilical vein endothelial cells
LLVA: Low-luminance visual acuity
logMAR: Logarithm of the minimum angle of resolution
MeSH: Medical Subject Headings
MMP2/9: Matrix metalloproteinase-2 and -9
MnSOD: Manganese superoxide dismutase
ND4: NADH dehydrogenase subunit 4
nAMD: Neovascular age-related macular degeneration
OCT: Optical coherence tomography
OIR: Oxygen-induced retinopathy
PEDF: Pigment epithelium-derived factor
PERG: Pattern electroretinography
RGC: Retinal ganglion cell
RNFL: Retinal nerve fiber layer
rAAV: Recombinant adeno-associated virus
RPGR: Retinitis pigmentosa GTPase regulator
scAAV: Self-complementary adeno-associated virus
SMD: Standardized mean difference
sFLT-1: Soluble fms-like tyrosine kinase-1
SYRCLE: Systematic Review Centre for Laboratory Animal Experimentation
TgIGF-I: Transgenic insulin-like growth factor 1
TIMP3: Tissue inhibitor of metalloproteinases 3
uPA: Urokinase-type plasminogen activator
uPAR: Urokinase plasminogen activator receptor
VA: Visual acuity
VEGF: Vascular endothelial growth factor
