# Supplementary material for: Ocular gene therapy targeting retinal angiogenesis and vascular leakage: translational and clinical evidence from a systematic review and meta-analysis
Source: Angiogenesis. 2026 Aug 31;29(4):66. doi: 10.1007/s10456-026-10050-y (PMC13529909; doi:10.1007/s10456-026-10050-y)
Supplement: Supplementary file 1 [file 10456_2026_10050_MOESM1_ESM.pdf]

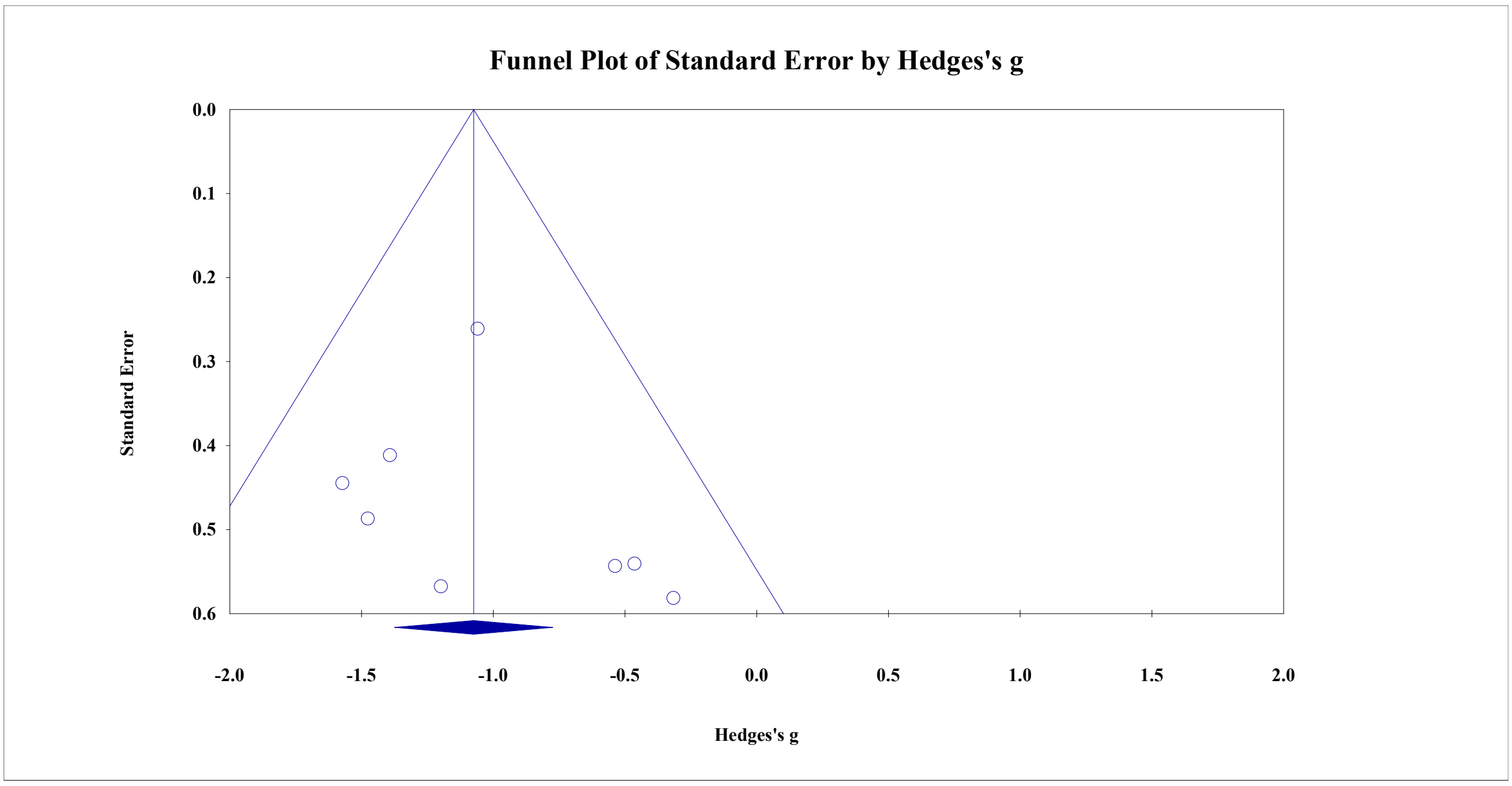

Supplementary Figure 1. Funnel plot assessing publication bias for neovascularisation outcomes in preclinical studies.

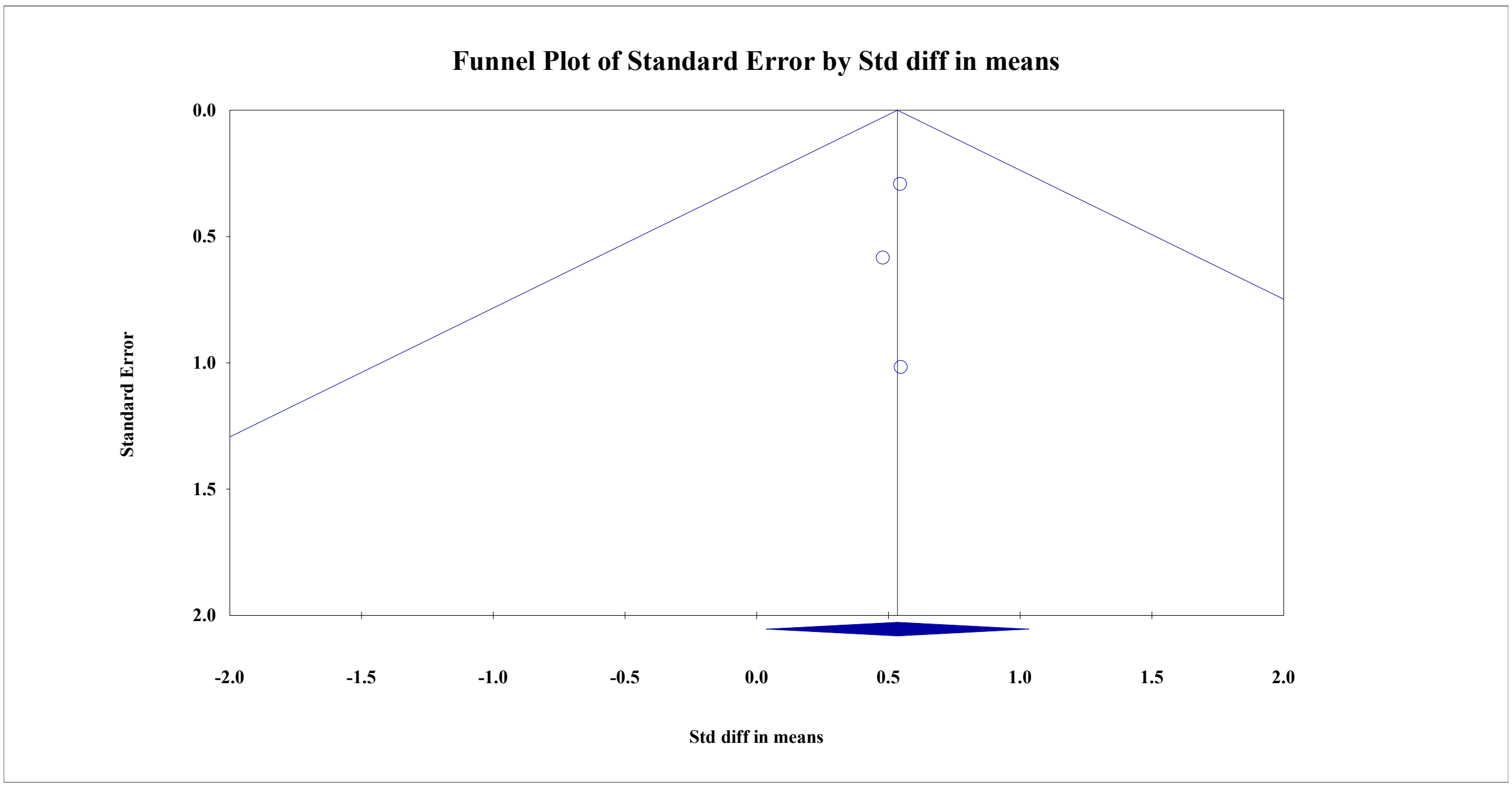

Supplementary Figure 2. Funnel plot assessing publication bias for vascular leakage outcomes in preclinical studies.

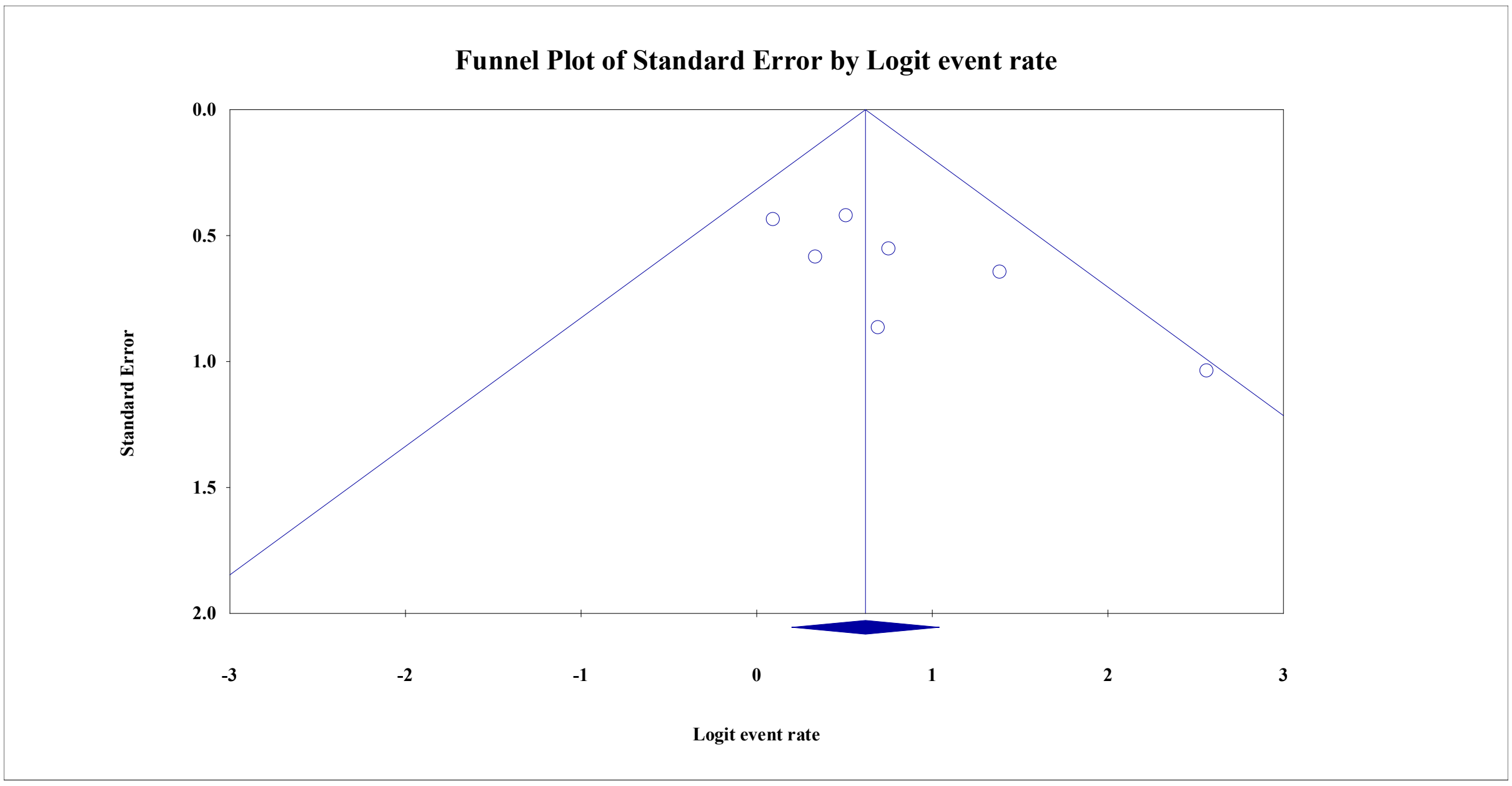

Supplementary Figure 3. Funnel plot assessing publication bias for Anti-VEGF injection burden

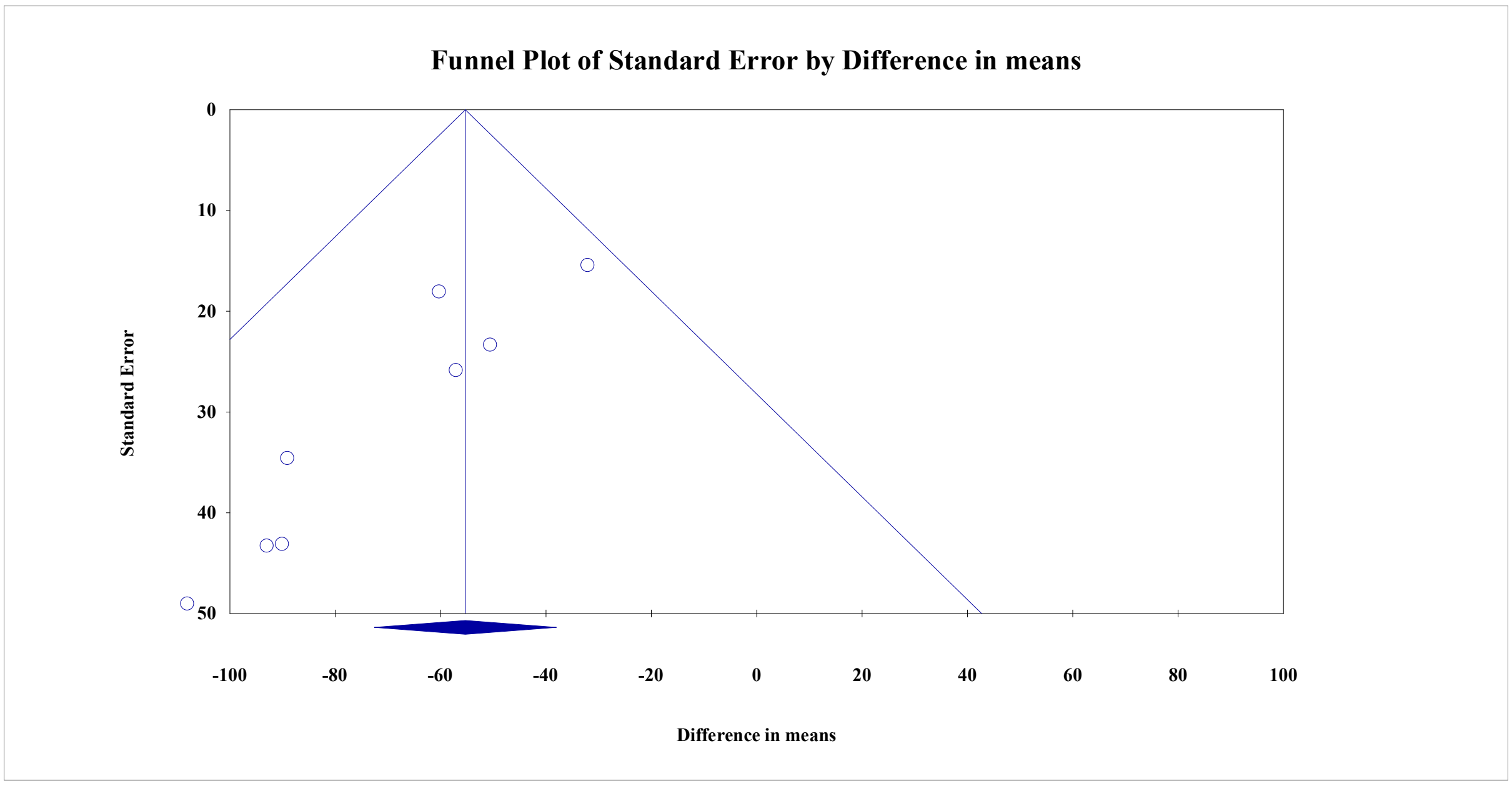

Supplementary Figure 4. Funnel plot assessing publication bias for CST
